# Evaluation of effectiveness and safety of pharmacist independent prescribers in care homes: cluster randomised controlled trial

**DOI:** 10.1136/bmj-2022-071883

**Published:** 2023-02-14

**Authors:** Richard Holland, Christine Bond, David P Alldred, Antony Arthur, Garry Barton, Linda Birt, Jeanette Blacklock, Annie Blyth, Stamatina Cheilari, Amrit Daffu-O'Reilly, Lindsay Dalgarno, James Desborough, Joanna Ford, Kelly Grant, Bronwen Harry, Helen Hill, Carmel Hughes, Jacqueline Inch, Vivienne Maskrey, Phyo Myint, Nigel Norris, Fiona Poland, Lee Shepstone, Maureen Spargo, David Turner, Laura Watts, Arnold Zermansky, David Wright

**Affiliations:** 1 Leicester Medical School, University of Leicester, Leicester, UK; 2 Institute of Applied Health Sciences, University of Aberdeen, Aberdeen, UK; 3 School of Healthcare, University of Leeds, Leeds, UK; 4 School of Health Sciences, University of East Anglia, Norwich, UK; 5 Norwich Medical School, University of East Anglia, Norwich, UK; 6 School of Pharmacy, University of East Anglia, Norwich, UK; 7 Geriatric Medicine, Addenbrooke's Hospital, Cambridge, UK; 8 Norwich Clinical Trials Unit, University of East Anglia, Norwich, UK; 9 Stow Healthcare Ltd, Bury St. Edmunds, UK; 10 School of Pharmacy, Queen’s University Belfast, Belfast, UK; 11 School of Education and Lifelong Learning, University of East Anglia, Norwich, UK; 12 School of Healthcare, University of Leicester, Leicester, UK

## Abstract

**Objective:**

To estimate the effectiveness, cost effectiveness (to be reported elsewhere), and safety of pharmacy independent prescribers in care homes.

**Design:**

Cluster randomised controlled trial, with clusters based on triads of a pharmacist independent prescriber, a general practice, and one to three associated care homes.

**Setting:**

Care homes across England, Scotland, and Northern Ireland, their associated general practices, and pharmacy independent prescribers, formed into triads.

**Participants:**

49 triads and 882 residents were randomised. Participants were care home residents, aged ≥65 years, taking at least one prescribed drug, recruited to 20 residents/triad.

**Intervention:**

Each pharmacy independent prescriber provided pharmaceutical care to approximately 20 residents across one to three care homes, with weekly visits over six months. Pharmacy independent prescribers developed a pharmaceutical care plan for each resident, did medicines reviews/reconciliation, trained staff, and supported with medicines related procedures, deprescribing, and authorisation of prescriptions. Participants in the control group received usual care.

**Main outcomes measures:**

The primary outcome was fall rate/person at six months analysed by intention to treat, adjusted for prognostic variables. Secondary outcomes included quality of life (EQ-5D by proxy), Barthel score, Drug Burden Index, hospital admissions, and mortality. Assuming a 21% reduction in falls, 880 residents were needed, allowing for 20% attrition.

**Results:**

The average age of participants at study entry was 85 years; 70% were female. 697 falls (1.55 per resident) were recorded in the intervention group and 538 falls (1.26 per resident) in the control group at six months. The fall rate risk ratio for the intervention group compared with the control group was not significant (0.91, 95% confidence interval 0.66 to 1.26) after adjustment for all model covariates. Secondary outcomes were not significantly different between groups, with exception of the Drug Burden Index, which significantly favoured the intervention. A third (185/566; 32.7%) of pharmacy independent prescriber interventions involved medicines associated with falls. No adverse events or safety concerns were identified.

**Conclusions:**

Change in the primary outcome of falls was not significant. Limiting follow-up to six months combined with a small proportion of interventions predicted to affect falls may explain this. A significant reduction in the Drug Burden Index was realised and would be predicted to yield future clinical benefits for patients. This large trial of an intensive weekly pharmacist intervention with care home residents was also found to be safe and well received.

**Trial registration:**

ISRCTN 17847169.

## Introduction

The need to improve prescribing and processes surrounding medicines in care homes (long term care facilities) is internationally recognised.^1^ ^2^ A large scale UK based observational study in 2009 identified that 70% of care home residents experienced drug errors daily.^2^ The authors identified the need for one individual to assume central responsibility for medicines management in care homes.^2^ In response, the UK government called for suitable interventions to tackle the problem.^3^ However, interventions to improve medicines management within care homes, usually involving either pharmacists or doctors providing medication reviews, have limited evidence for clinical effectiveness.^4^

Reasons postulated for the lack of evidence include variability in trial design, lack of development of interventions, and poor selection of outcome measures.^4^ Hence, people have called for “high-quality cluster-randomised controlled trials testing multidisciplinary interventions that measure well defined, important resident-related outcomes.”^4^ A 2019 systematic review reported falls as the only patient centred outcome to be improved as a result of pharmacist interventions in care homes.^5^ Equally, the usual model of care, which is based on pharmacists making recommendations for drug changes, creates extra work for doctors and, as the recommendations are frequently not enacted, represents a waste of pharmacists’ time.^6^

UK legislative changes in 2006 enabled accredited pharmacists to prescribe independently,^7^ and they can operate autonomously when assuming a central medicines optimisation role—for example, within care homes. Pharmacist independent prescribers (PIPs) are able to identify pharmaceutical needs and initiate, change, or monitor medicines without secondary authorisation. Several studies have shown the effectiveness of PIPs in non-care home contexts,^8^ ^9^ ^10^ but no evaluation has been done in care homes. Implementation of pharmacist prescribing generally in the UK has been variable, with relatively little evaluation of clinical effectiveness.^11^ ^12^ However, with the expansion of clinical pharmacy in English general practice and the requirement now for newly qualified pharmacists to be trained as prescribers,^13^ pharmacist prescribing is being implemented nationally and includes prescribing for care home residents.^12^

In 2015 the UK National Institute for Health and Care Research funded a programme of research (the Care Homes Independent Pharmacist Prescriber Study or CHIPPS) to evaluate this model of care. The programme followed the Medical Research Council’s guidance on development and evaluation of complex interventions,^14^ with extensive stakeholder engagement,^15^ selection of outcome measures,^16^ development of a training programme to enhance fidelity,^6^ ^17^ and a feasibility study in four UK locations that showed acceptability of the service and confirmed feasibility of recruitment.^18^ These culminated in this cluster randomised controlled trial with an internal pilot,^19^ ^20^ which was designed to assess the clinical effectiveness and cost effectiveness (to be reported elsewhere) of PIPs providing pharmaceutical care within care homes compared with usual care.^21^

## Methods

### Study design

This cluster randomised controlled trial was conducted in geographical areas associated with the grant holders—namely, East of England, Grampian (Scotland), Northern England, and Northern Ireland. The trial protocol,^19^ summarised below, started with an internal pilot. Recruitment and delivery of the intervention ran from March 2018 to March 2020.

### Participants and inclusion/exclusion criteria

We recruited triads (clusters) of a general practice, a PIP, and care home(s) providing approximately 20 residents each. All the PIPs needed to be UK accredited prescribers and were excluded if they already provided a similar service to the recruited care home or had a conflict of interest through employment with the supplying community pharmacy. We included general practices if they managed sufficient care home residents to support recruitment of 20 eligible participants. We included care homes if they provided care primarily to adults aged over 65 years and were associated with a participating general practice. We excluded them if their residents already received regular, drug focused review services (defined as monthly or more frequently) or if they were under formal investigation by a regulator. We included residents who were under the care of a participating general practice, aged over 65 years, permanently resident in a participating care home, taking at least one regular medicine, and able to provide (directly or via an appropriate representative) informed consent/assent. We excluded residents if they were receiving end-of-life care or participating in another study.

### Triad and resident identification and recruitment

We used invitation packs, containing invitation letters, information sheets, and consent forms, to recruit PIPs and general practices, identified using local networks. Consenting general practitioners then approached up to three care homes to enable recruitment of approximately 20 residents. Care home managers distributed invitation packs, signed by the general practitioner, to potential residents or appropriate third parties (for example, next of kin) for those residents lacking capacity to consent.

### Randomisation and blinding

Randomisation was done (one-to-one ratio for intervention and control groups) at the triad level, stratified by the four geographical areas, by using a web based electronic system integrated into the centrally maintained REDCap database.^22^ Researchers responsible for recruitment of general practitioners, care homes, and residents were blinded to allocation during the recruitment phase but unblinded thereafter. Incidents of blinding being broken for research associates were recorded. PIPs allocated to the intervention arm were trained for their role after randomisation and broke blinding for care homes and general practices once they started their formal interactions.

### Intervention

See supplementary materials for the CHIPPS protocol and the service specification (appendix 2 in the protocol).^19^ We developed the PIP service specification with stakeholders and identified potential barriers to implementation, with a clearly defined PIP role and effective communication deemed key to success.^15^ PIPs received study specific training for their role over a six week period after randomisation,^17^ and we provided them with materials needed for their role at this stage, such as PowerPoint slides from their training and STOPP/START criteria for medication review.^23^ We developed this training programme on the basis of a systematic review,^6^ along with stakeholder engagement, expert panel consensus, and feasibility testing.^17^ The training programme involved face-to-face training on managing medicines for older people with complex needs, a personal development framework, and mentorship. Subsequent to training, PIPs were assessed by a general practitioner and a pharmacist mentor, and their competencies were signed off.^24^ To allow for completion of training and sign-off, we standardised time zero at six weeks post-randomisation.

The PIPs visited the care homes to do medication reviews and optimise therapy for all participating residents, and they created pharmaceutical care plans to record their activity and provide a plan for future activity. Pharmaceutical care plans also allowed the PIP’s actions/plans to be recorded for the care home and the resident’s general practitioner. Additionally, the PIPs provided general support for improving care home processes for ordering medicines (to minimise opportunity for missed doses), administration of medicines (to reduce administration errors), medicines reconciliation when residents transferred between settings (to minimise opportunity for transcription errors), and staff training (to optimise requests for new medicines such as antipsychotics, laxatives, and analgesics). The nature and extent of delivery of each element of the intervention was individualised for the care home by the PIPs, each of whom was allocated four hours a week to manage an average of 20 residents over six months.

PIPs were responsible for updating residents’ records within care homes and general practices and for communicating changes to the supplying pharmacist. They decided on the most appropriate methods for communicating changes to general practices and care homes—that is, orally, in writing, or in person, depending on the activity. The intervention was tailored to context (for example, training to care home staff) and was delivered according to need.

### Control

Participants in the control group received usual general practitioner led care, which could range from visits purely in response to individual requests to regular weekly sessions to provide more proactive care. Pharmacist provision could range from provision of medicines only (by a community pharmacist) to three, six, or 12 monthly visits by primary care based pharmacists doing medication reviews. Few, if any, of these reviews would have involved pharmacists actively prescribing, as opposed simply to providing advice to the general practitioner. PIPs recruited and trained within the trial had no interaction with control homes.

### Outcomes

We developed a core outcome set for trials of the effectiveness of prescribing in care homes to inform selection of outcomes for this trial,^16^ in combination with data from our feasibility study.^18^ From that work, we selected a primary outcome of fall rate/person over six months, as recorded in care homes’ falls records, which are required by regulators. Secondary outcomes (at six months unless stated otherwise) selected were resident’s (by proxy) quality of life (EQ-5D-5L)^25^ at three and six months with responses converted into a utility score, with 0 indicating death and 1 full health^26^; proxy modified physical functioning score (Barthel) in which 0 is most dependent and 20 least dependent^27^; Drug Burden Index, a measure of anticholinergic and sedative drug exposure, collected via medication data recorded by the general practitioner, in which higher scores indicate greater anticholinergic potential and increased risk of drug related morbidity^28^; hospital admissions over six months’ follow-up, collected from general practice records supplemented by care home records; mortality; and health service use and associated costs. Our CHIPPS logic model also informed this final outcome selection (see supplementary materials). Data collection started in September 2018 and concluded in July 2020.

### Safety

We defined serious adverse events as unexpected admission to hospital, death related to the study intervention, or both. Suspected serious adverse events were both reported prospectively by general practitioners and identified retrospectively by the trial manager through proactive monthly contact with care homes. The resident’s general practitioner assessed serious adverse events for causality and association with the PIP intervention. In addition, a dedicated email address was provided to all care home staff to report concerns. Finally, a study geriatrician assessed a 20% random sample of pharmaceutical care plans (weighted towards earlier trial stages) for clinical appropriateness and safety.

### Sample size calculation

On the basis of the fall rate observed in the CAREMED study (to assess effectiveness of multidisciplinary medication review in care homes),^29^ we aimed for 880 participants (440/arm). This number was sufficient to provide 80% statistical power to detect a 21% difference in fall rate from 1.50 to 1.18 per resident over six months, using a two sided 5% significance level, and included an assumed attrition of 20%. We assumed that the study would consist of 44 clusters, with a mean of 20 participants in each, and an intra-class correlation coefficient of ≤0.05. The estimate of a reduction in falls was half that suggested by another UK care home pharmacist intervention.^30^

### Statistical analysis

We used a frequentist approach, with a two sided 5% statistical significance level for hypothesis testing, providing estimates of between group differences and corresponding 95% confidence intervals. The primary analysis was done on an intention-to-treat basis (that is, participants were analysed within their allocated group, rather than by actual treatment received), with a per protocol analysis also completed for participants deemed to have received the PIP intervention as intended. We anticipated that the primary outcome would follow a Poisson distribution, but the data proved to best fit a negative binomial model, which we used instead. We estimated parameters by using a generalised estimating equation approach, to account for the clustered design, with an offset included for length of follow-up. Length of follow-up varied from participant to participant owing to death or dropout.

The final primary outcome model included baseline fall rate, key prognostic variables (defined as baseline values of Drug Burden Index,^28^ Barthel score,^27^ Charlson Comorbidity Index,^23^ and home status (nursing/residential)), with group as a fixed factor. We also included an offset of logarithm of follow-up time to allow inclusion of information from participants lost to follow-up before six months. We used an analogous generalised estimating equation model for secondary and sensitivity analyses, with an appropriate change to the link and error term, depending on the nature of the outcome of interest.

We used a Cox proportional hazards regression model (time from consent to death or otherwise censored) for mortality analyses, in which we used robust sandwich estimates of standard errors to adjust for clustering within care homes. We used SAS v.9.4 for analyses. The trial was overseen by a Data Monitoring Committee and an over-arching Trial Steering Committee.

### Process evaluation

Following Medical Research Council guidance,^9^ we conducted a mixed methods process evaluation, including quantitative and qualitative data collection at the end of each six month implementation period. This is reported elsewhere.^31^

### Patient and public involvement

We worked with our local Public and Patient in Research (PPIRES) group from the outset, with feedback received on the original project idea from PPIRES members and subsequent involvement in the grant application as it developed. With support from our care home expert (HH), we visited a home to conduct a focus group with residents, listening to their views about the project and what was needed from a resident’s perspective. Residents had no concerns about a pharmacist prescriber looking after their medicines, but they wanted involvement in all decisions. Training on this was incorporated into the final intervention.

To enhance research effectiveness, we recruited four patient and public involvement members with an interest in care homes through PPIRES: two on the management group and two for the independent steering committee. Patient and public involvement members were family carers of people with complex conditions requiring polypharmacy. One had previous experience of working in care homes. We sought the views of patient and public involvement members involved in the management group at all points during the study, and one wrote an article about this.^32^ They reviewed the participants’ information leaflets, consent forms, training materials, and qualitative data, so as to include their different perspectives on our findings. Patient and public involvement members also reviewed abstracts and papers before publication. In addition, we engaged with the Patients Association to support dissemination of findings and help to organise our final dissemination event. Patient and public involvement collaborators were involved in dissemination through a public facing conference for general practitioners, pharmacists, and care home staff.

## Results

**Fig 1:**
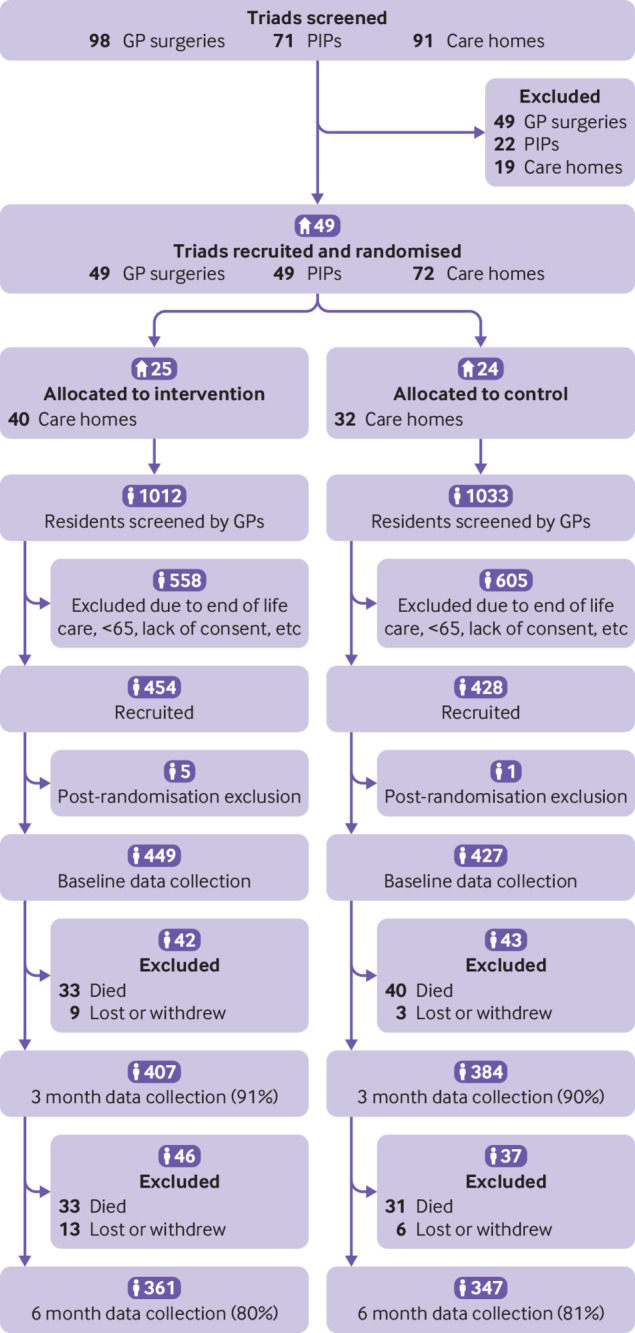
Consort diagram for cluster randomised controlled trial. GP=general practitioner; PIP=pharmacist independent prescriber

We recruited 49 triads (49 general practices, 49 PIPs, and 72 care homes) (fig 1). In total, between 15 February 2018 and 10 September 2019, 25 triads, including 449 residents, were randomly allocated to the intervention and 24 triads, including 427 residents, to control. Almost all losses to follow-up at six months (137/168; 82%) were due to deaths of residents. Excluding those, primary outcome data were available for 96% of participants. One care home (11 residents) closed during the study, and three of the 25 intervention PIPs did not deliver the full service, one of whom did not deliver the intervention at all. Five cases of unblinding of researchers to care home allocation were reported.

Table 1 shows the baseline characteristics of the groups. Most variables were similar between groups, but the control group had rather more male residents (33% *v* 28%) and a greater proportion in nursing home care (59% *v* 42%). The intervention group had higher Barthel scores (8.34 *v* 7.07; ie, greater independence) and a greater rate of falls, with mean falls in the previous 90 days of 0.78 compared with 0.57 for controls.

**Table 1:** Trial groups at baseline. Values are numbers (percentages) unless stated otherwise

Characteristics	Intervention residents (n=449)	Control residents (n=427)	Overall residents (n=876)
Mean (SD) age at consent, years	85.1 (7.7)	85.4 (7.6)	85.3 (7.7)
Sex:			
Male	125 (28)	141 (33)	266 (30.4)
Female	324 (72)	286 (67)	610 (69.6)
Consent:			
Participant	59 (13)	51 (12)	110 (12.6)
Consultee	390 (87)	376 (88)	766 (87.4)
Care home status:			
With nursing	188 (42)	250 (59)	438 (50.5)
Residential only	256 (58)	174 (41)	430 (49.5)
Missing	5	3	8
No of drugs:			
Median (IQR)	6 (4-9)	6 (4-9)	6 (4-9)
Range	1-19	1-19	1-19
Missing	2	4	6
Falls in previous 90 days:			
Median (IQR)	0 (0-1)	0 (0-1)	0 (0-1)
Range	0-30	0-18	0-30
Mean (SD)	0.78 (2.30)	0.57 (1.43)	0.68 (1.93)
Hospital admissions in previous 90 days:			
Median (IQR)	0 (0-0)	0 (0-0)	0 (0-0)
Range	0-3	0-2	0-3
Mean (SD)	0.09 (0.33)	0.07 (0.26)	0.08 (0.30)
Mean (SD) Barthel score	8.34 (5.78)	7.07 (5.77)	7.74 (5.81)
Missing	10	35	45
Mean (SD) Drug Burden Index	0.72 (0.75)	0.70 (0.69)	0.71 (0.72)
Missing	5	2	7
Mean (SD) Charlson Comorbidity Index	5.94 (1.84)	5.98 (1.52)	5.96 (1.69)
Missing	5	6	11
Mean (SD) EQ-5D self-utility score*	0.49 (0.37)	0.33 (0.36)	0.41 (0.37)
Missing	396	377	773
Mean (SD) EQ-5D proxy utility score*	0.31 (0.35)	0.29 (0.37)	0.30 (0.36)
Missing	34	55	89

IQR=interquartile range; SD=standard deviation.

* EQ-5D utility scores set to zero for participants who died

Table 2 shows outcome data. The median follow-up time was 198 days in the intervention arm and 197 days in the control arm. In all, 697 falls (1.26 per resident) were recorded in the intervention group at six months and 538 (1.55 per resident) in the control group. After adjustment for all model covariates, we found no significant difference between groups, with a rate ratio of 0.91 (95% confidence interval 0.66 to 1.26). Per protocol analysis of the primary outcome did not change the outcome. The intra-class correlation was 0.051 when we included all model covariates, very close to our assumed intra-class correlation of 0.05.

**Table 2:** Falls at six months: summary

Falls	Intervention (n=449)	Control (n=427)	Rate ratio (95% CI); P value (model 1)*	Rate ratio (95% CI); P value (model 2)†
Total falls	697	538	-	-
Follow-up (person days)	79 803	76 904	-	-
Crude fall rate/year	3.19	2.56	1.00 (0.73 to 1.36); 0.99	0.91 (0.66 to 1.26); 0.58
Range	0-59	0-27	-	-
Interquartile range	0-2	0-1	-	-
Median	0	0	-	-

CI=confidence interval.

* Adjusted for falls at baseline (in 90 days before enrolment); all 876 participants included, but only 844 had non-zero follow-up time.

† Adjusted for falls at baseline, Barthel score, Drug Burden Index, Charlson Comorbidity Index, and care home status (nursing/residential); 812 participants included.

In total, 66 (15%) deaths were reported in the intervention group compared with 71 (17%) in the control group, with a mean time to death of 109 and 103 days, respectively. The Cox’s proportional hazards model found no evidence of an intervention effect (adjusted hazard ratio 0.93, 95% confidence interval 0.64 to 1.35; P=0.68) (see survival analysis figure in supplementary materials).

Table 3 shows secondary outcome results including Drug Burden Index, hospital admissions, and Barthel scores. Drug Burden Index results showed an effect in the intervention group, with an improvement from 0.72 to 0.66, whereas values in the control group worsened from 0.70 to 0.73; the ratio of Drug Burden Index scores at six months between intervention and control was 0.83 (0.74 to 0.92; P<0.001). No other secondary outcome showed a statistically significant difference.

**Table 3:** Secondary outcomes at six months

Outcome	Intervention (n=449)	Control (n=427)	Rate ratio* (95% CI); P value	Rate ratio (95% CI); P value (fully adjusted comparison)†
Hospital admissions per person:				
** **Median (IQR)	0 (0-0)	0 (0-0)	-	-
** **Range	0-4	0-3	-	-
** **Mean (SD)	0.19 (0.50)	0.18 (0.47)	0.98 (0.66 to 1.46); 0.93	0.90 (0.61 to 1.32); 0.57
Barthel score:				
** **Mean (SD)	8.12 (5.84)	6.46 (5.66)	1.19 (0.96 to 1.49); 0.12	1.20 (0.96 to 1.49); 0.11
** **Missing	113	110	-	-
Drug Burden Index:				
** **Mean (SD)	0.66 (0.74)	0.73 (0.69)	0.83 (0.74 to 0.92); <0.001	0.83 (0.74 to 0.92); <0.001
** **Missing	10	9	-	-

CI=confidence interval; IQR=interquartile range; SD=standard deviation.

* Comparison adjusted for baseline values of main variable only.

† Comparison adjusted for baseline value of main variable, Barthel score, Charlson Comorbidity Index, home status, and Drug Burden Index.

Table 4 shows the EQ-5D results. Across both arms the level of missing EQ-5D data at baseline, three months, and six months was respectively 10.2% (see table 1), 14.2%, and 11.4% (see table 4). EQ-5D scores were very similar at baseline between groups and changed little through follow-up, with small, statistically non-significant differences at three and six months. We identified no safety concerns from review of pharmaceutical care plans or independent assessment of serious adverse events, of which none recorded was related to the intervention. Table 5 summarises the types of interventions undertaken by the PIPs. In total, 668 interventions were recorded including 566 clinical interventions, of which 185 (32.7%) involved medications related to falls risk; 148 (26.1%) of them reduced that risk.^31^

**Table 4:** EQ-5D proxy outcomes at three and six months

EQ-5D proxy utility score	Intervention (n=449)	Control (n=427)	Absolute difference* (95% CI); P value	Absolute difference (95% CI); P value (fully adjusted comparison)†
3 months:				
** **Mean (SD)‡	0.28 (0.35)	0.28 (0.35)	−0.017 (−0.073 to 0.039); 0.56	−0.043 (−0.092 to 0.006); 0.08
** **Missing	77	47	-	-
6 months:				
** **Mean (SD)‡	0.26 (0.35)	0.21 (0.33)	0.030 (−0.021 to 0.080); 0.25	0.042 (−0.043 to 0.052); 0.86
** **Missing	53	47	-	-

CI=confidence interval; SD=standard deviation

Self-reported data are available from the authors but were collected from only 6.5% of resident participants.

* Comparison adjusted for baseline values of EQ-5D only.

† Comparison adjusted for baseline value of EQ-5D, Barthel score, Charlson Comorbidity Index, home status, and Drug Burden Index.

‡ EQ-5D utility scores set to zero for participants who died.

**Table 5:** Number and type of pharmacist independent prescriber interventions per patient

Category	No (%) interventions (n=668)
Interventions per resident (average)	1.8
Technical intervention	99 (14.8)
Educational intervention	3 (0.4)
Clinical intervention	566 (84.7)
Type of clinical intervention:	(n=566)
Drug discontinuation/dose reduction	379 (67.0)
Start new drug	60 (10.6)
Change drug	49 (8.7)
Dose increase	26 (4.6)
Monitoring	52 (9.2)

## Discussion

Introducing PIPs to care homes did not reduce falls in care home residents over a six month follow-up period. However, the anticholinergic/sedative “burden” (Drug Burden Index) of medicines taken by care home residents was reduced by almost a fifth compared with usual care, suggesting that effective deprescribing occurred. All other secondary outcomes, after adjustment for baseline differences, showed no significant difference.

### Strengths and limitations of study

In terms of validity, this was a large trial, involving 72 care homes, 49 PIPs and general practices, and 876 residents. We recruited to target, so we had sufficient power to test our hypothesis of a 21% decrease in falls. As this was a cluster trial, we removed the potential for contamination affecting performance of different care homes managed by the same general practice. Follow-up of residents was thorough and complete, with primary outcome data available for 96% of residents at six months when deaths were excluded.

This trial was the culmination of a five year programme grant during which the team, assiduously following Medical Research Council guidance,^14^ developed the intervention and PIP training in careful consultation with a wide array of key stakeholders to ensure that PIPs were maximally effective.^17^ We also did a feasibility study to ensure that PIPs were appropriately prepared; residents, care homes, and general practitioners could be recruited; and outcome data could be collected efficiently.^18^

In hindsight, benefits from drug related interventions, particularly deprescribing, take time to be realised, so a six month intervention and follow-up period may have been insufficient. The intervention development phase also highlighted a need for PIPs to be part of the general practice team; however, during recruitment, insufficient general practice based pharmacists were available to allow us to recruit solely from that pool. That three (12%) PIPs failed to deliver the full intervention was also unfortunate, but, as this was a pragmatic study, this is consistent with what would happen in everyday practice. Blinding care homes to their group status was also not possible, and researchers may have been aware of that status when collecting follow-up data.

We developed a core outcome set to support selection of the most valid outcome measures for this group,^16^ which we tested in the feasibility study.^18^ We identified number of falls as the most suitable primary outcome, as it is readily obtainable and objective, has low potential for missing data, is resident centred, is relevant to a wide range of morbidities, and is a direct and indirect consequence of drug effects. However, many other factors contribute to residents falling, such as their condition, their environment, and their general care.^33^ Furthermore, although criteria are available for defining and recording a fall in a care home,^34^ these are not universally adopted in practice, and no standardised template for recording a fall is available. These differences should average out across as large a study as this one, but this “random misclassification” had the potential to reduce evidence of effectiveness.

In our process evaluation,^31^ we identified that just over a quarter of residents experienced an intervention that had the potential to reduce the likelihood of falls; however, in a small proportion, interventions had the potential to increase that likelihood. Both reductions and increases in the risk of falls are rarely immediate consequences of drug changes; rather, the likelihood of falling over time is modified. Thus, a 12 month or even 24 month follow-up may have been more desirable.

Anticholinergic/sedative burden is associated with increased mortality, falls, hip fractures, frailty, and reduced quality of life.^35^ ^36^ ^37^ ^38^ Thus, the significant reduction in Drug Burden Index observed should predict improved outcomes for residents. However, data on Drug Burden Index and risk have been based on a minimum 12 months of observation.^36^ ^37^ ^38^ ^39^ Again, this study’s six month follow-up may have been unlikely to fully realise clinical improvements.

### Comparison with other studies

Our results contrast with the evidence for effectiveness of PIPs in other contexts.^8^ ^9^ ^10^ These studies were, however, based in younger populations and focused on one disease area. Care home residents are, by definition, complex and frequently on a steep downward trajectory with respect to quality of life. Consequently, results are not comparable.

### Generalisability

The broad inclusion criteria for residents mean that our findings are highly generalisable and relevant across the UK care home sector, and also internationally, although few other countries yet have pharmacists with full prescribing rights.^40^ ^41^

### Conclusions

This large, rigorously conducted, cluster randomised controlled trial, testing a pharmacist independent prescriber regularly visiting care homes to manage residents’ pharmaceutical care, showed that this was a safe, well received intervention,^31^ which decreased anticholinergic/sedative prescribing. Although this would be expected to realise future clinical benefits, the intervention showed no improvement in our primary outcome of falls. We identified integration of PIPs into care homes and general practices as being necessary to enhance the effectiveness of the intervention. Equally, care home triallists have yet to identify a fully appropriate patient centred outcome that is able to measure clinically relevant changes across a wide range of residents.

## What is already known on this topic

Medicines management for care home residents is in need of significant improvement, with observational studies indicating that >50% of residents experience ​medication errors daily
Interventions to improve medicines management in care homes have shown limited effectiveness
UK pharmacists can prescribe independently, but no study to date has assessed the effectiveness of pharmacist independent prescribers (PIPs) in care homes

## What this study adds

An intervention introducing PIPs to visit care homes weekly for approximately four hours was safe and welcomed by care home staff and general practitioners
Introducing PIPs to care homes did not reduce falls in care home residents over a six month follow-up period
PIPs reduced the Drug Burden Index, suggesting that they can successfully improve residents’ medication, which may yield health benefits to residents beyond six months

## Data Availability

De-identified participant data, including primary, secondary, and patient reported outcome data, interview transcripts, prescription data, protocol, and statistical analysis plan, are available on request from DW (d.j.wright@leicester.ac.uk), LS (l.shepstone@uea.ac.uk), and Norfolk and Waveney Integrated Care Board (NWICB) research office (nwicb.RandDoffice@nhs.net). Reuse of the CHIPPS dataset will be made available on reasonable request for the purpose of improving patient care in health and social care and will be subject to completion of a data sharing agreement between NWICB, the University of East Anglia, and the third-party organisation.

## Web extra

Extra material supplied by authors

Web appendix: Supplementary materials

## Notes

Contributors: All authors reviewed the paper before submission. DW led the whole project jointly with RH. RH and CB led the main trial, on which this paper is based. RH was involved in conceptualisation, funding acquisition, methodology, project administration, writing the original draft, and reviewing and editing the manuscript. CB, DA, GB, FP, and AA were involved in conceptualisation, funding acquisition, methodology, project administration, and reviewing and editing the manuscript. CH and NN were involved in conceptualisation, funding acquisition, data curation, methodology, project administration, and reviewing and editing the manuscript. LS was involved in conceptualisation, formal analysis, funding acquisition, methodology, project administration, and reviewing and editing the manuscript. LB was involved in data curation, methodology, project administration, and reviewing and editing the manuscript. JB and SC were involved in data curation and reviewing and editing the manuscript. AB and LW were involved in data curation, project administration, resources, and reviewing and editing the manuscript. ADOR, MS, and VM were involved in data curation, project administration, and reviewing and editing the manuscript. LD, BH and JI were involved in data curation, project administration, and reviewing and editing the manuscript. JD was involved in conceptualisation, funding acquisition, methodology, and reviewing and editing the manuscript. JF was involved in project administration and reviewing and editing the manuscript. KG was involved in formal analysis and reviewing and editing the manuscript. HH was involved in conceptualisation, funding acquisition, methodology, project administration, and reviewing and editing the manuscript. PKM was involved in funding acquisition, project administration, and reviewing and editing the manuscript. DT was involved in formal analysis, project administration, and reviewing and editing the manuscript. AZ was involved in conceptualisation, funding acquisition, methodology, and reviewing and editing the manuscript. DW was involved in conceptualisation, funding acquisition, data curation, methodology, resources, writing the original draft, and reviewing and editing the manuscript. DW and RH are the guarantors. The corresponding author attests that all listed authors meet authorship criteria and that no others meeting the criteria have been omitted.

The lead authors (the manuscript’s guarantors) affirm that this manuscript is an honest, accurate, and transparent account of the study being reported; that no important aspects of the study have been omitted; and that any discrepancies from the study as planned (and, if relevant, registered) have been explained.

Dissemination to participants and related patient and public communities: We engaged with the Patients Association to support dissemination of findings and help to organise our final dissemination event. Patient and public involvement collaborators were involved in this dissemination event through a public facing conference for general practitioners, pharmacists, and care home staff.

Provenance and peer review: Not commissioned; externally peer reviewed.
