# Supplementary material for: Evaluation of effectiveness and safety of pharmacist independent prescribers in care homes: cluster randomised controlled trial
Source: BMJ. 2023 Feb 14;380:e071883. doi: 10.1136/bmj-2022-071883 (PMC9926330; doi:10.1136/bmj-2022-071883)

CHIPPS

Care Homes Independent Pharmacist Prescribing Service (CHIPPS): A cluster randomised controlled trial to determine both its effectiveness and cost-effectiveness.

Work Package 6: RCT with internal pilot: a definitive randomised controlled trial

Version	[v5.1]
Date	23 October 2019
Sponsor	South Norfolk Clinical Commissioning Group
NRES #	[insert MREC number]

Authorisation: Co-Chief Investigator

Name	Professor Richard Holland
Signature	[insert wet signature]

Authorisation: Co-Chief Investigator

Name	Professor Christine Bond
Signature	[insert wet signature]

Authorisation: Programme Chief Investigator

Name	Professor David Wright

Signature	[insert wet signature]
Date	[insert date]

Authorisation: Sponsor Representative

Name	Ms Clare Symms
Signature	[insert wet signature]
Date	[insert date]

Authorisation: NCTU Director

Name	Professor Ann Marie Swart
Signature	[insert wet signature]
Date	[insert date]

Authorisation: Trial Statistician

Name	Professor Lee Shepstone
Signature	[insert wet signature]
Date	[insert date]

Table of Contents

1 Administrative information 1
1.1 Compliance 1
1.2 Sponsor 1
1.3 Structured study summary 2
1.4 Roles and responsibilities 5
1.4.2 Role of study sponsor and funders 5
1.4.3 Location Research Staff 5
1.4.4 Programme Management Group; assumes role of Trial Management Group 5
1.4.5 Programme Steering Committee 9
1.4.6 Data Monitoring Committee 10
2. Study Timeline 11
3 Abbreviations 12
4 Glossary 13
5 Introduction 14
5.1 Background and Rationale 14
5.1.1 Explanation for choice of comparators 15
5.2 Objectives 15
5.3 Study Design 16
6 Methods 16
6.1 Site Selection 16
6.1.1 Study Setting 16
6.1.2 Location/Investigator Criteria 16
6.1.2.1 Resourcing at location 16
6.1.3 Withdrawal from Study 16
6.1.3.1 PIPs 17
6.1.3.2 GP practices 17
6.1.3.3 Care home associated with the consented GP practice. 17
6.1.3.4 Residents of the consented GP practice who are resident in the care home. 17
6.2 Site approval and activation 17
6.3 Participants 18

6.3.1 Eligibility Criteria for Prescribing Independent Pharmacist (who will deliver the intervention) ... 18
6.3.1.1 Inclusion criteria: ... 18
6.3.1.2 Exclusion criteria: ... 18
6.3.2 Eligibility Criteria for GP Practices ... 19
6.3.2.1 Inclusion criteria: ... 19
6.3.3 Eligibility Criteria for Care Homes ... 19
6.3.3.1 Inclusion criteria: ... 19
6.3.3.2 Exclusion criteria: ... 19
6.3.4 Eligibility Criteria for care home residents ... 19
6.3.4.1.1 Inclusion criteria: ... 19
6.3.4.1.2 Exclusion criteria: ... 20
6.4 Interventions ... 20
6.4.1 Compliance and Adherence ... 22
6.5 Outcomes ... 22
6.5.1 Further Outcomes ... 23
6.5.1.2 Process Outcomes ... 23
6.5.1.3 Health Economic ... 23
6.6 Figure 1. Participant Timeline ... 24
6.6.1 Early Stopping of Follow-up ... 25
6.6.2 Participant Transfers ... 25
6.6.3 Loss to Follow-up ... 25
6.6.4 Study Closure ... 25
6.7 Randomisation ... 25
6.8 Sample Size ... 27
6.9 Recruitment and Retention ... 27
6.9.1 Recruitment ... 27
6.9.1.1 PIP recruitment ... 29
6.9.1.2. GP practice recruitment ... 29
6.9.1.3 Care home recruitment ... 29
6.9.1.4 Resident recruitment ... 29
6.9.2 Retention of participants ... 32
6.10 Data Collection, Management and Analysis ... 32

6.10.1 Data Collection Methods ... 32
6.10.2 Data Management ... 32
6.10.3 Non-Adherence and Non-Retention ... 33
6.10.4 Statistical Methods ... 33
6.10.4.1 Statistical Analysis Plan ... 33
6.10.4.2 Additional Analyses – Subgroup ... 34
6.10.5 Analysis Population and Missing Data ... 34
6.10.5.1 Economic evaluations ... 34
6.11 Data Monitoring ... 35
6.11.1 Data Monitoring Committee ... 35
6.11.1.2 Interim Analyses ... 35
6.11.2 Data Monitoring for Harm ... 35
6.11.2.1 Safety reporting of Serious Adverse Events ... 36
6.11.2.1.1 Causality ... 36
6.11.2.1.2 Notification of SUSARs and Safety Concerns to the NCTU ... 37
6.11.2.1.3 Notification of SUSARs and Safety Concerns to the DMC ... 37
6.11.3 Quality Assurance and Control ... 37
6.11.3.1 Risk Assessment ... 37
6.11.3.2 Monitoring of pharmaceutical care plans (PCPs) ... 38
6.11.3.3 Central Monitoring at NCTU ... 38
6.11.3.4 On-site Monitoring ... 38
6.11.3.4.1 Direct access to participant records ... 38
6.11.3.5 Study Oversight ... 38
6.11.3.5.1 Trial Management Group ... 39
6.11.3.5.2 Programme Steering Committee ... 39
6.11.3.5.3 Study Sponsor ... 39
7 Ethics and Dissemination ... 39
7.1 Research Ethics Approval ... 39
7.2 Other Approvals ... 39
7.3 Protocol Amendments ... 39
7.4 Consent or Assent (in the case of residents without capacity) ... 40
7.4.1 Process Evaluation ... 41
7.5 Confidentiality ... 42

7.6 Declaration of Interests ... 42

7.7 Indemnity ... 43

7.8 Finance ... 43

7.9 Archiving ... 43

7.10 Access to Data ... 43

7.11 Ancillary and Post-study Care ... 43

7.12 Publication Policy ... 43

7.12.1 Study Results ... 43

7.12.2 Authorship ... 43

7.12.3 Reproducible Research ... 43

8 Ancillary Studies ... 44

9 Protocol Amendments ... 44

10 References ... 44

11 Appendices ... 45

Appendix 1. The PIP - further information for CHIPPS ... 45

Appendix 2. Service Specification ... 46

Appendix 3. List of data accessed from residents' records ... 51

Appendix 4. Inclusion of participants who lack capacity ... 55

Appendix 5. List of NCTU documents ... 61

1 Administrative information

This document was constructed using the Norwich Clinical Trials Unit (NCTU) Protocol template Version 3. It forms the protocol for Work Package 6 (WP6) of the CHIPPS programme of work sponsored by NHS South Norfolk Clinical Commissioning Group and co-ordinated by NCTU.

This document provides information about procedures for entering participants into the trial, and provides sufficient detail to enable: an understanding of the background, rationale, objectives, trial population, intervention, methods, statistical analyses, ethical considerations, dissemination plans and administration of the trial; replication of key aspects of trial methods and conduct; and appraisal of the trial's scientific and ethical rigour from the time of ethics approval through to dissemination of the results. Every care has been taken in drafting this protocol, but corrections or amendments may be necessary. These will be circulated to registered investigators in the trial. Sites entering participants for the first time should confirm they have the correct version through a member of the trial team at NCTU.

NCTU supports the commitment that protocols for its trials adhere to the SPIRIT guidelines. This protocol therefore includes the Standard Protocol Items: Recommendations for Interventional Trials (SPIRIT) 2012 Statement for protocols of clinical trials [1]. The SPIRIT Statement Explanation and Elaboration document [2] can be referred to, or a member of NCTU Protocol Review Committee can be contacted for further detail about specific items.

1.1 Compliance

The trial will be conducted in compliance with the approved protocol, the Declaration of Helsinki (2008), the principles of Good Clinical Practice (GCP), the UK Data Protection Act, GDPR, Adults with Incapacity Act (Scotland) 2000, Mental Capacity Act England 2005, and the National Health Service (NHS) Research Governance Framework for Health and Social Care (RGF) and other national and local applicable regulations. Agreements that include detailed roles and responsibilities will be in place between participating sites and NCTU.

1.2 Sponsor

NHS South Norfolk Clinical Commissioning Group is the sponsor and has delegated responsibility for the management of the CHIPPS trial to the Trial Co-Chief Investigators and NCTU. Queries relating to sponsorship of this study should be addressed to the Sponsor's Representative.

1.3 Structured study summary

Primary Registry and Study Identifying Number	ISRCTN – 17847169
Date of Registration in Primary Registry	15/12/2017
Secondary Identifying Numbers	Programme Grant Registration number: RP-PG-0613-10018
Source of Monetary or Material Support	NIHR PGfAR
Sponsor	NHS South Norfolk Clinical Commissioning Group
Public Queries	Annie Blyth Research Fellow/Senior Programme Coordinator, UEA a.blyth@uea.ac.uk<br><br>Vivienne Maskrey Research Fellow/Senior Programme Coordinator, UEA v.maskrey@uea.ac.uk
Contact for Scientific Queries	Richard Holland, WP6 Co-CI<br>Head of Medical School and Professor of Public Health Medicine<br>Leicester Medical School<br>Centre for Medicine<br>University of Leicester<br>University Road<br>Leicester. LE1 7RH<br>0116 252 3022<br>rch23@leicester.ac.uk<br><br>Christine Bond, WP6 Co-CI<br>Emeritus Professor of Primary Care<br>Division Applied Health Sciences<br>Polwarth Building<br>Foresterhill<br>University of Aberdeen<br>Aberdeen. AB25 2AY<br>01224 553973<br>c.m.bond@abdn.ac.uk
Public Title	CHIPPS RCT
Scientific Title	CHIPPS Work Package 6: RCT with internal pilot: a definitive randomised controlled trial.
Countries of Recruitment	England, Scotland, Northern Ireland
Health Condition(s) or Problem(s) Studied	The overall aim of the Programme Grant is to test if making Pharmacist Independent Prescribers (PIPs) part of the care home team, working alongside general practitioners, could

	improve quality of care for those over 65 years old resident in care homes.
Intervention(s)	Intervention<br>At intervention care home(s), Pharmacist Independent Prescribers (PIPs) working in collaboration with the relevant GP(s), will assume responsibility for the medicines management of a mean of 20 care home residents living in one or more care homes associated with the GP practice.<br><br>The PIP will deliver the intervention, according to the CHIPPS Service Specification developed in previous work packages, by:<br>• reviewing participant medication and developing and implementing a pharmaceutical care plan<br>• assuming prescribing responsibilities<br>• supporting systematic ordering, prescribing and administration processes within each care home, GP practice and supplying pharmacy where needed<br>• providing training to staff in care home and GP practice<br>• communicating with GP practice, care home, supplying community pharmacy and study team<br><br>Control<br>At each control care home, medicine management will be according to usual practice, in which the GP(s) has responsibility for the medicines management of care home residents living in one or more care home(s) associated with the GP practice; pharmacy provision will also be according to usual practice in that area

Key Inclusion and Exclusion Criteria	**CARE HOME RESIDENTS:**<br>**Inclusion criteria**<br>Residents who are:<br>• under the care of the GP practice<br>• 65 years or over<br>• currently prescribed at least one regular medication<br>• able to provide informed consent/assent, or have a consultee (England and Northern Ireland) or Welfare Power of Attorney (WPoA) (Scotland) able to provide informed consent<br>• permanently resident in care home (not registered for respite care/temporary resident)<br><br>**Exclusion criteria**<br>Residents who are/have:<br>• currently receiving end of life care (equivalent to yellow [{stage C)] of the Gold Standards Framework prognostic indicator)<br>• additional limitations on their residence (e.g. held securely)<br>• participating in another intervention research study<br><br>Inclusion and Exclusion criteria relating to the GPs, PIPs and Care Homes are detailed in Section 6.3
Study Type	Clustered randomised controlled trial, with internal pilot
Date of First Enrolment	December 2017: first recruitment of PIP/GP practice/care home triads<br><br>February 2018: first recruitment of care home residents
Target Sample Size	880 care home residents (160 of whom will comprise the internal pilot)
Outcome(s)	The following outcomes will be measured at the time points indicated<br><br>Primary Outcome<br>• fall rate per person at 6 months<br><br>Secondary Outcomes<br>• proxy EQ-5D-5L (quality of life) at baseline, 3 months and 6 months<br>• face to face self-reported EQ-5D-5L (only applicable for participants with capacity) at baseline, 3 months and 6 months

	• proxy Barthel Index (physical functioning) at baseline and 6 months<br>• fall rate per person at 3 months<br>• health-service utilisation (and associated costs) at baseline and 6 months<br>• mortality<br>• hospitalisations<br>• Drug Burden Index (DBI)

1.4 Roles and responsibilities

These membership lists are correct at the time of writing; please see terms of reference documentation in the SMF for current lists.

1.4.2 Role of study sponsor and funders

Name	Affiliation	Role and responsibilities
Clare Symms	Sponsor representative	Contributed to study design and management

1.4.3 Location Research Staff

Name	Affiliation	Role and responsibilities
Dr Maureen Spargo	Queens University Belfast	Research Fellow (RF) in Belfast with responsibility for all aspects of study delivery in that location.<br><br>Dr Maureen Spargo will be on Maternity Leave from May 2018 until March 2019; Mairead McGrattan has been employed to cover this period.
Dr Amrit Daffu-O'Reilly	University of Leeds	Research Fellow (RF) in Yorkshire with responsibility for all aspects of study delivery in that location.
Ms Jackie Inch	University of Aberdeen	Research Fellow (RF) in Grampian with joint responsibility for all aspects of study delivery in that location (job share with Frances Notman).
Dr Frances Notman	University of Aberdeen	Research Fellow (RF) in Grampian with joint responsibility for all aspects of study delivery in that location (job share with Jackie Inch).
Ms Jeanette Blacklock	UEA	Research Associate in Norfolk to undertake recruitment, data collection, data entry and administrative tasks as required in that location.

1.4.4 Programme Management Group; assumes role of Trial Management Group

Name	Affiliation	Role and responsibilities
Dr David Alldred	University of Leeds	Location PI for Leeds, and Programme grant holder.

		Design and manage study. Attend trial management meetings. Prepare study report. Contribute to development of papers for dissemination. Attend dissemination events. Oversee the delivery of the study in Yorkshire and manage Leeds Research Fellow. Recruit local medical practices and care homes. Organise and attend local dissemination events.
Professor Anthony Arthur	University of East Anglia	Programme grant holder. Secondary care expert; nursing expert. Design and manage study. Attend trial management meetings. Prepare study report. Contribute to development of papers for dissemination. Attend dissemination events.
Professor Garry Barton	Norwich CTU	Programme grant holder. Health Economics expert. Design and manage study. Attend trial management meetings. Prepare study report. Contribute to development of papers for dissemination. Attend dissemination events. Provide health economics oversight throughout. Manage health economics RA.
Ms Annie Blyth	University of East Anglia	Senior Programme Co-ordinator with joint responsibility for overseeing the delivery of the programme (job share with Vivienne Maskrey). Attend trial management meetings.
Professor Christine Bond	University of Aberdeen	Co-CI for trial, Location PI for Aberdeen and Programme grant holder. Design and manage study. Attend trial management meetings. Prepare study report. Contribute to development of papers for dissemination. Attend dissemination events. Oversee the delivery of study and manage the location research staff in Grampian. Recruit local medical practices and care homes.
Dr James Desborough	University of East Anglia	Programme grant holder. Pharmacy practice expert. Design and manage study. Attend trial management meetings. Prepare study report. Contribute to development of papers for dissemination. Attend dissemination events. Contribute to delivery of PIP training. Support local delivery of study in Norfolk.
Dr Joanna Ford	Norfolk and Norwich	Study geriatrician.<br>Programme grant holder. Design and manage study. Attend trial management meetings. Prepare study

	University Hospital	report. Contribute to development of papers for dissemination. Attend dissemination events. Review pharmaceutical care plans and related records.
Ms Christine Handford	Public and Patient Involvement in Research Panel, Norfolk and Suffolk (PPIRES)	Attend trial management meetings and contribute to: design and management of the study, development of participant information resources, undertake/analyse the research, contribute to the reporting of the study and dissemination of research findings. Attend dissemination events.
Ms Helen Hill	Care Home Manager	Care homes expert. Attend trial management meetings and contribute to: design and management of the study, development of participant information resources, undertake/analyse the research, contribute to the reporting of the study and dissemination of research findings.
Professor Richard Holland	University of Leicester	Co-CI for programme. Co-CI for trial, Programme grant holder, Design and manage study, Attend trial management and Programme Steering Committee. Review progress reports for NIHR. Oversee development of service specification. Prepare study report, Prepare papers for dissemination. Attend dissemination events.
Professor Carmel Hughes	Queens University, Belfast	Location PI for Belfast, Programme grant holder. Design and manage study. Attend trial management meetings. Prepare study report. Contribute to development of papers for dissemination. Organise and attend dissemination events. Oversee delivery of study in Belfast and manage Belfast location research staff. Recruit local medical practices and care homes.
Ms Vivienne Maskrey	University of East Anglia	Senior Programme Co-ordinator with joint responsibility for overseeing the delivery of the programme (job share with Annie Blyth). Attend trial management meetings.
Ms Kate Massey	PPIRES	Attend trial management meetings and contribute to: design and management of the study, developing participant information resources, undertaking/analysing the research, contribute to the reporting of the study and dissemination of research findings. Attend dissemination events.
Professor Phyo Myint	University of Aberdeen	Study geriatrician.<br>Programme grant holder. Design and manage study. Attend trial management meetings. Prepare study report. Contribute to development of papers for dissemination. Attend dissemination events. Review pharmaceutical care plans and related records.

Professor Nigel Norris	University of East Anglia	Programme grant holder. Education and training expert. Design and manage study. Attend trial management meetings. Prepare study report. Contribute to development of papers for dissemination. Attend dissemination events. Assist with development of training package.
Professor Fiona Poland	University of East Anglia	Programme grant holder. Qualitative research expert. Design and manage study. Attend trial management meetings. Prepare study report. Contribute to development of papers for dissemination. Attend dissemination events. Responsible for providing qualitative analysis support.
Professor Lee Shepstone	Norwich Clinical Trials Unit	Programme grant holder. Statistical analysis expert. Design and manage study. Attend trial management meetings. Prepare study report. Contribute to development of papers for dissemination. Attend dissemination events.
Mr Ian Small	Norwich Clinical Commissioning Group	Programme grant holder. Primary care pharmacy practice expert. Design and manage study. Attend trial management meetings. Prepare study report. Contribute to development of papers for dissemination. Attend dissemination events.
Professor Ann Marie Swart	Norwich CTU	Clinical trials unit director with oversight of CTU activities, clinician, epidemiologist and clinical trials expert.
Ms Clare Symms	NHS South Norfolk Clinical Commissioning Group	Attend trial management meetings and contribute to management of the study, with responsibility for NHS costs management.
Ms Joanna Williams	Norwich Clinical Trials Unit	Clinical Trial Manager responsible to CTU Director and Co-CIs for delivery of the trial.
Professor David Wright	University of East Anglia	Co-CI for over all programme and location PI for Norfolk.<br><br>Programme grant holder. Design and manage study. Attend trial management meetings. Prepare study report. Contribute to development of papers for dissemination. Organise and attend dissemination events. Manage Norfolk location research staff and study administrator. Regularly visit sites to oversee project delivery. Chair trial management meetings. Attend Programme Steering Committee steering committee. Prepare progress reports for NIHR. Prepare study report, Prepare papers for dissemination. Disseminate findings nationally and internationally.

Dr Arnold Zermansky	University of Leeds	Programme grant holder. Primary care and GP practice expert. Design and manage study. Attend trial management meetings. Prepare study report. Contribute to development of papers for dissemination. Attend dissemination events.

1.4.5 Programme Steering Committee

Name	Affiliation	Role and responsibilities
Professor Tim Peters	University of Bristol	Chair, Independent
Dr Keith Ridge	Chief Pharmaceutical Officer; England DoH	Independent
Professor Sarah Lewis	University of Nottingham	Statistician, Independent
Professor Rose Marie Parr	Chief Pharmaceutical Officer, Scotland Scottish Government	Independent
Dr Mark Timoney	Chief Pharmaceutical Officer, Northern Ireland;<br>DoH	Independent
Professor Christian Mallen	Keele University	GP, Independent
Dr Judy Henwood	Norfolk & Suffolk Primary and Community Care Research Office	Research Design Lead, Norfolk & Suffolk Primary and Community Care Research Office
Ms Joyce Groves	Lay member (PPIRes)	Lay member (PPIRes), Independent
Ms Elaine Bounds	Lay member (PPIRes)	Lay member (PPIRes), Independent
Ms Jennifer Griffiths	Back up Lay member (PPIRes)	Back up Lay member (PPIRes) Independent
Professor David Wright	UEA	Co-CI of programme
Professor Richard Holland	University of Leicester	Co-CI of programme/Co-CI of trial
Professor Christine Bond	University of Aberdeen	Co-CI of trial

1.4.6 Data Monitoring Committee

Name	Affiliation	Role and responsibilities
Professor John Campbell	University of Exeter Medical School	Professor of General Practice and Primary Care Director, trialist
Professor Sandra Eldridge	Blizard Institute, Barts and The London School of Medicine and Dentistry, Queen Mary University of London	Professor of Biostatistics, statistician
Professor Bryony Dean Franklin	UCL School of Pharmacy / Imperial College Healthcare NHS Trust	P Professor of Medication Safety, pharmacist
Professor Lee Shepstone	Norwich Clinical Trials Unit	Programme Statistician<br>(attending to represent the trial)
Joanna Williams	Norwich Clinical Trials Unit	Clinical Trial Manager<br>(attending to represent the trial)
Professor Richard Holland<br>or<br>Professor Christine Bond	University of Leicester<br><br>University of Aberdeen	Co-CI of programme / Co-CI of trial<br><br>Co-CI of trial<br>(attending to represent the trial)

2. Study Timeline

Diagram 1. Pilot and RCT Timeline overview

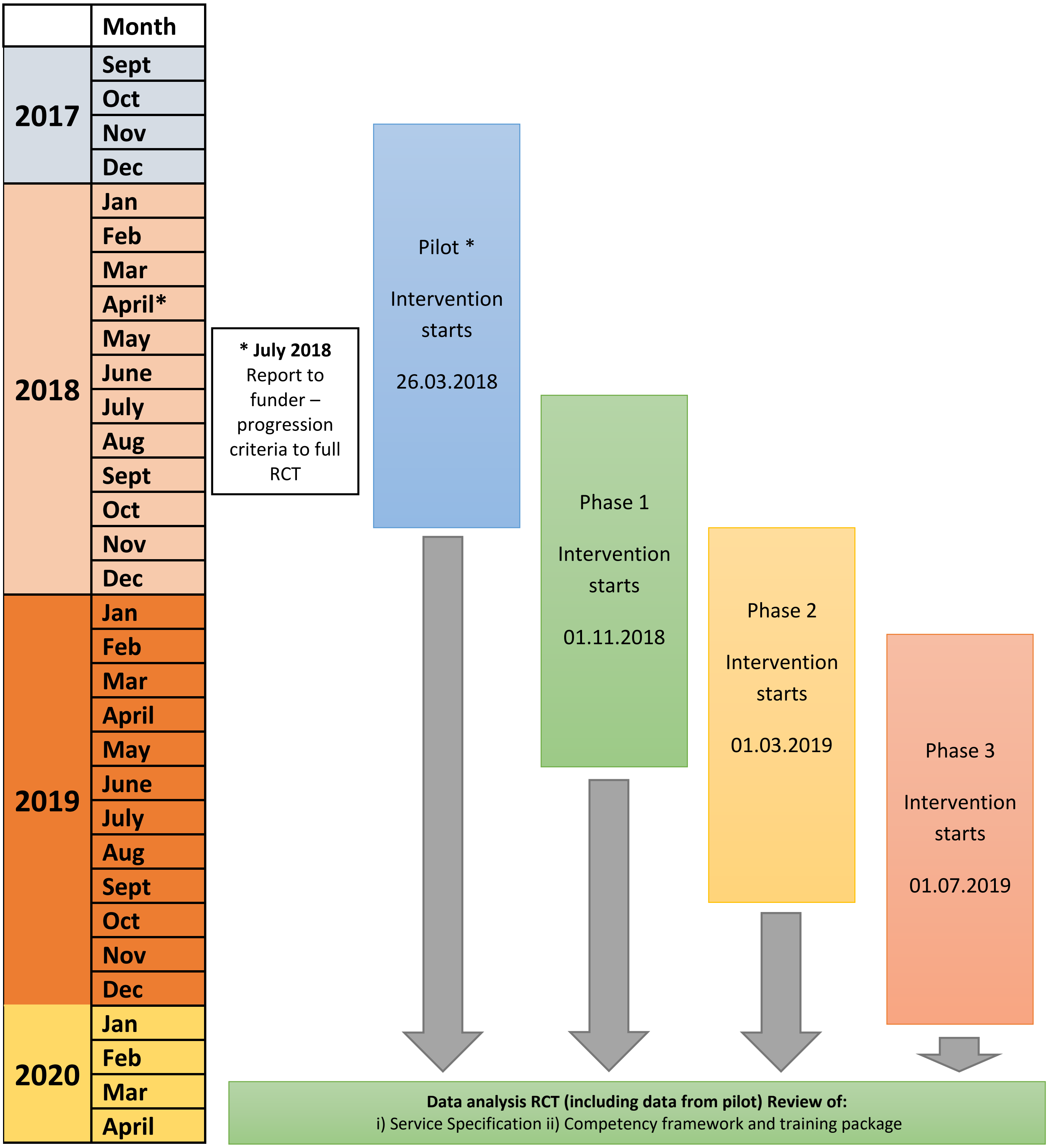

3 Abbreviations

ADE	Adverse Drug Event
ADR	Adverse Drug Reaction
AE	Adverse Event
CCG	Clinical Commissioning Group
CHUMS	Care Homes' Use of Medicines Study (CHUMS) 2009
CI	Chief Investigator
Co-CI	Co-Chief Investigator
CRF	Case Report Form
CTA	Clinical Trial Authorisation
DMC	Data Monitoring Committee
DSUR	Development Safety Update Report
GCP	Good Clinical Practice
HB	Health Board
HRA	Health Research Authority
ICH	International Conference on Harmonisation
ICO	Information Commissioner's Office
IRAS	Integrated Research Application System
LCRN	Local Clinical Research Network
LPOA	Lasting Power of Attorney *(this applies only in England & NI)*
NCTU	Norwich Clinical Trials Unit
NICE	National Institute for Health and Care Excellence
NIHR	National Institute for Health Research
PCP	Pharmaceutical Care Plan
PI	Principal Investigator
PIP	Pharmacist Independent Prescribers
PIS	Participant Information Sheet
PMG	Programme Management Group
PoA	Power of Attorney *(this applies in England & NI only)*
PPI	Patient and Public Involvement
Norfolk & Suffolk PPIRes	Public and Patient Involvement in Research, Norfolk and Suffolk
PSC	Programme Steering Committee
PSS	Person Shaped Support
QA	Quality Assurance
QALY	Quality-adjusted life-year
QC	Quality Control
QMMP	Quality Management and Monitoring Plan
RA	Research Associate
R&D	Research and Development
RCT	Randomised Controlled Trial
REC	Research Ethics Committee
SAE	Serious Adverse Event
SAP	Statistical Analysis Plan

SAR	Serious Adverse Reaction
SMF	Study Master File
SPC	Summary of Product Characteristics
SSA	Site Specific Approval
SSI	Site Specific Information
SUSAR	Suspected Unexpected Serious Adverse Reaction
TMF	Trial Master File
ToR	Terms of Reference
WP	Work Package
WPoA	Welfare Power of Attorney *(this applies only in Scotland)*

4 Glossary

EQ-5D-5L	Standardised instrument for measuring quality of life
Barthel Index	Standardised instrument for measuring physical function
Drug Burden Index	Tool used to measure cumulative exposure to anticholinergic and sedative medications in older adults
Local research Office	Four regional centres. Each location has a local PI, a local researcher and a local research office(s).
Pharmaceutical Care Plan	A structured resident record detailing medication history, current medication, indications for use of drugs and recommended changes
Pharmacist Independent Prescribers (PIP)	A registered pharmacist with additional formal qualifications allowing them to prescribe under the non-medical prescriber regulations
Research Site	GP Practice and Associated Care Home/s
Setting	Primary care (participating GP-PIP-Care home triads)
STOPP START	Screening tool used to identify instances of potentially inappropriate prescribing in older adults

5 Introduction

5.1 Background and Rationale

In 2012, care homes in the UK provided accommodation for almost half a million residents in beds registered for either residential or nursing care. The transfer from one's own home to a facility which provides 24-hour care is usually as a result of being unable to live independently, mainly due to a deterioration in health. Consequently, care home residents are generally frail, have multiple morbidities and are prescribed a significant number of regular medicines. Unfortunately, age-related complex morbidity renders them particularly vulnerable to medication problems and errors. The Care Quality Commission identifies the management of medicines as one area of care in care homes that regularly requires review and continues to fall below the expected standards.

The landmark UK-based Care Homes Use of Medicines Study (CHUMS) published in 2009 observed 256 residents in 55 care homes. Almost 70% of residents experienced at least one medication error on any given day. One hundred residents (39.1%) were identified as having one or more prescribing errors. For 20%, no strength or route of medicine was specified; for almost one quarter, a medicine was deemed to be unnecessary. Dose/strength errors accounted for 14.4% of all errors; occasions when a prescribed medicine had not been given, accounted for 11.8%, of all errors. Out of 218 potentially harmful medicines which required biochemical monitoring, 32 (14.7%) had an error. This was most often a failure to request blood tests. Fifty-seven (22.3%) residents had a total of 116 administration errors (i.e. errors on the drug round), nearly half of which were omissions and more than one fifth the wrong dose. Carers were observed using inappropriate techniques when administering medicines such as inhalers. Problems with medicines ordering and stock holding led to omissions. Hospital discharge letters were also criticised for being unclear, delayed, missing or not adequately incorporated into the residents' clinical records. The researchers noted that the main method of communication regarding medicines was the medication administration chart and this was often inaccurate.

Many of these medication-related problems were also reported in a systematic review by Alldred et al [3] which considered interventions to optimise prescribing for older people in care homes. Problems highlighted were prescription of medicines that were no longer indicated, medicines which interacted with concurrent medication, sub-optimal doses, inadequate monitoring and inappropriate duration. The inappropriate prescription of anti-psychotic medicines in care homes is well documented and is known to be related to poor quality of life, falls and increased mortality. Other medicines with potential for long-term harm that are known to be prescribed inappropriately in care homes are benzodiazepines, non-steroidal anti-inflammatory drugs and proton pump inhibitors. Consequently, effective interventions are needed to monitor and discontinue therapy. Inadequate monitoring can result in sub-optimal dosing, over-treatment or the unintentional treatment of side effects that have been incorrectly identified as a new symptom that requires further treatment.

The CHUMS report proposed that the fundamental failing in care homes was the lack of a healthcare professional with overall continuing responsibility for medicines management and recommended that a pharmacist should adopt this role working with a lead general practitioner (GP) within each home. The Department of Health (DH) Immediate Action Alert arising from CHUMS required primary care organisations, GPs and community pharmacy contractors to establish effective joint working strategies to address the identified concerns. The resultant predominant model of care is that of a pharmacy team undertaking full medication reviews in care homes on a yearly or biannual basis. Two recent Cochrane reviews conducted by Hughes and Alldred [3, 4] suggest that this model is sub-optimal and more effective approaches to medicines optimisation in this population are required.

Recent changes in UK legislation, enabling suitably trained pharmacists to prescribe, provides an opportunity for pharmacist independent prescribers (PIPs) to assume the proposed central role in the care home environment. Evidence from the UK, led by Bond and involving Wright and Holland, suggests that pharmacist independent prescribers can prescribe safely and provide patient benefit. It would also be similar to that mandated in the USA, whereby a pharmacist is required to be an integral part of the care home team where they develop, implement and monitor individualised medicines-focussed (pharmaceutical) care plans. However they are not responsible for prescribing. (Appendix 1)

We propose that a suitable model for care homes is a PIP, who would assume responsibility for appropriate medicines management, monitoring and authorising repeat prescriptions, and overall management of medicines. Such a PIP would use pharmaceutical care plans (PCPs) to communicate prescribing decisions and plans between members of the care team. PCPs state the indication for each medicine, monitoring requirements (efficacy and side effects), review date and additional relevant information e.g. related policies or guidance on administration method (for a full list of all the data that will be collected in the PCP. Please see Appendix 3). The PIP would develop PCPs when establishing the service, liaising with the care home staff, residents and GP where necessary. They would then review the PCP on a monthly basis to confirm efficacy, ensure that adverse drug reactions are identified and managed, the ongoing need for therapy is considered and that monitoring is requested in a timely manner. PCPs would be regularly updated, reviewed and integrated within medical practice and care home records. The PCP would be a detailed record of all resident-related medication activities undertaken by the PIP and act as an aide memoire for the provision of future care. To address concerns identified within the CHUMs study, the PIP could additionally assume responsibility for managing transfer of medicines information between care locations, observe medication administration and actively ensure that stock levels are adequate to prevent missed doses.

Before introducing this innovative model of care, in addition to determining its optimal content, we anticipate a large number of logistical and professional barriers may need to be overcome. We have therefore developed a programme of work comprising six work packages (WPs). This protocol relates solely to WP6 (RCT with internal pilot) which was informed by work undertaken during the earlier work packages (using the literature and views of stakeholders (care home managers, staff, residents and relatives, GPs and pharmacists), development of a needs based PIP training, and a non-randomised feasibility study) to determine the final service specification and training package for the PIPs.

5.1.1 Explanation for choice of comparators

The comparator will be usual GP led care, as we are wanting to evaluate the effect of adding a prescribing pharmacist to the current team. We recognise that the current team may already include a pharmacist, but experience tells us that although this is common practice it rarely consists of visits occurring more than twice a year, and is unlike the intensive approach proposed in this study. Any medical practices which employ pharmacists to provide services to care homes of similar intensity to that which we propose will be excluded. Randomisation will be at practice level rather than home level due to concerns regarding contamination which may occur if two homes were in the same practice and one received the intervention whilst the other didn't. It is not appropriate to randomise at resident level as the intervention is designed to affect care home culture and therefore control residents would not be immune to its effects. There would also be a risk of contamination within homes and practices.

5.2 Objectives

The objectives for WP6 RCT with internal pilot are:

- to use an embedded (internal) pilot study to confirm:
    - feasibility of recruiting sufficient GP practices, PIPs, care homes and residents
    - availability of data for primary outcome at 3 months
    - there are no intervention related safety concerns
- if the pilot is successful (see page 21) to deliver a full RCT to:
    - describe the clinical effectiveness of the intervention: pharmacist independent prescribers assuming responsibility for medicines management of elderly residents in care homes
    - to estimate the cost-effectiveness of the intervention

5.3 Study Design

This is a cluster randomised controlled trial, which will include an internal pilot study, conducted in care homes for older people and associated GP practices.

6 Methods

6.1 Site Selection

The study sponsor has overall responsibility for location, site and investigator selection and has delegated this activity to the programme's Project Management Group.

6.1.1 Study Setting

The setting is primary care involving participating GP-PIP-Care home triads in the four study locations (geographical areas near the Universities of East Anglia, Leeds, Aberdeen and Queen's Belfast; hereinafter referred to just by the University identity).

6.1.2 Location/Investigator Criteria

All four participating locations) have been pre-identified in the programme grant application. Principal Investigators at each location are Co-Applicants on the programme grant, and each location has appropriately qualified and trained staff available to recruit study participants (GPs, PIPs, Care Homes, residents), collect and enter data and organise follow up.

No additional locations will be recruited. The Trial Manager will provide each location with a copy of this protocol, once it is approved by the relevant ethics committee.

6.1.2.1 Resourcing at location

The potential for recruiting the required number of care homes and suitable residents within the agreed recruitment period has been confirmed in the completed WP5 feasibility study and will be checked following the internal pilot.

Locations will complete a delegation of responsibilities log, and provide staff contact details.

Locations will have sufficient data management resources to allow prompt data return to NCTU.

6.1.3 Withdrawal from Study

The study involves four different participant types consenting or agreeing to participate, in the study. These are detailed below:

6.1.3.1 PIPs

If a PIP withdraws from the study, and a new PIP is not appointed within the 6 month study period, the care home and participating residents will be informed that the PIP intervention is no longer taking place, but that the data will be collected for individual resident participants at standard data collection times and may still be used in the final analysis.

6.1.3.2 GP practices

If a GP practice withdraws from the study before the care home has been recruited, then another GP practice will be recruited. If a GP practice withdraws, during the intervention period, it will not be replaced. All data collected up to that point will be kept and used according to the consent form and GP Agreement with the sponsor, both of which are signed by the GP.

6.1.3.3 Care home associated with the consented GP practice.

In the event a care home withdraws participation (or becomes ineligible e.g. following a regulatory inspection) within the first month of resident recruitment, the local research team will ask the GP practice to suggest another care home if possible. The care home will be made aware that data collected on the care home and residents of that care home to date cannot be erased and may still be used in the final analysis. Residents will be advised, by the research team, that their care home has withdrawn and they can no longer take part in the study.

6.1.3.4 Residents of the consented GP practice who are resident in the care home.

If a resident chooses to leave the study prematurely the primary reason for discontinuation will be determined and recorded, if at all possible. Withdrawn participants will not be replaced, unless they withdraw before the start of the intervention. Residents will be made aware (via the information sheet and consent form) that should they withdraw, the data collected to date cannot be erased and may still be used in the final analysis.

6.2 Site approval and activation

Recruitment will be done using recruitment triads; each triad will consist of one GP plus one PIP and at least one care home with capacity to recruit twenty residents.

A care home will be considered activated when the following has been completed:

- HRA (in England) or local R&D approval in Scotland and Northern Ireland secured
- Research Ethics Committee approval secured
- relevant local R&Ds informed and/or approval secured
- Stakeholder Agreements signed by the participating GP, and care home manager and the Sponsor's Representative
- Responsibilities Logs signed
- staff contact details agreed.

Once the care home is activated, recruitment of study residents may begin.

The site will conduct the study in compliance with the protocol as agreed by the Sponsor, approved by the relevant regulatory authorities, and given favourable opinion by the Research Ethics

Committee (REC). The PI or delegate will document and explain any deviation from the approved protocol, and communicate this to the study team at NCTU.

A list of activated sites may be obtained from the Trial Manager.

6.3 Participants

The eligibility criteria for this study have been carefully considered and are the standards used to ensure that only appropriate participants are entered. Participants not meeting the criteria will not be entered into the study for their safety and to ensure that the study results can be appropriately used to make future treatment decisions for other people in similar situations. No exceptions will be made to these eligibility criteria.

Residents will be considered eligible for enrolment in this study if they fulfil all the inclusion criteria and none of the exclusion criteria as defined below.

6.3.1 Eligibility Criteria for Prescribing Independent Pharmacist (who will deliver the intervention)

6.3.1.1 Inclusion criteria:

- registered as a pharmacist independent prescriber
- following CHIPPS study training, can demonstrate to their mentor and independent GP assessor competence to deliver service specification
- ability to work flexibly and commit a minimum of 16 hours a month to deliver the service for six months

6.3.1.2 Exclusion criteria:

- substantive employment with the community pharmacy (branch/store) which supplies medicines to the care home with which the PIP would work, to protect against conflict of interest
- already providing an intensive service to the care home, e.g. a monthly visit (or more frequently), and provision of intensive medication focused services

6.3.2 Eligibility Criteria for GP Practices

6.3.2.1 Inclusion criteria:

- GP practice must manage sufficient care home residents to support recruitment of the target of approximately 20 eligible participants[1].

6.3.3 Eligibility Criteria for Care Homes

6.3.3.1 Inclusion criteria:

- Care Quality Commission (CQC) in England, Care Inspectorate in Scotland or Regulation and Quality Improvement in Northern Ireland, registered specialism as caring for adults over 65
- primarily caring for residents over 65 years
- associated with a participating GP practice (i.e. one or more residents registered with a participating practice)

6.3.3.2 Exclusion criteria:

- care homes which receive regular (e.g. a monthly visit or more frequently), from a pharmacist, providing another intensive medication focused services
- care homes which receive regular (e.g. a monthly visit or more frequently), from another health care professional, providing another intensive medication focused services
- care homes which are currently under formal investigation with Care Quality Commission (CQC) in England, Care Inspectorate in Scotland or Regulation and Quality Improvement in Northern Ireland
- care homes that are participating in any other study likely to affect the outcome of the CHIPPS trial (e.g. Falls intervention study, Rehydration study, etc.)

6.3.4 Eligibility Criteria for care home residents

6.3.4.1.1 Inclusion criteria:

- under the care of the participating GP practice
- aged 65 years or over
- currently prescribed at least one regular medication
- they or their appropriate representative is/are able to provide informed consent/assent

[1] Based on earlier work, we anticipate a consent rate of 55% and so would expect to invite approximately 35 residents / GP practice

- permanently resident in care home (not registered for respite care/temporary resident)

6.3.4.1.2 Exclusion criteria:

- currently receiving end of life care, [equivalent to yellow (stage C) of the Gold Standards Framework prognostic indicator]
- have additional limitations on their residence (e.g. held securely)
- participating in another intervention research study

6.4 Interventions

The intervention will be delivered by trained PIPs for a period of six months. The intervention has already been tested in the earlier feasibility study and will involve the PIP, in collaboration with the care home resident's GP, assuming responsibility for managing the medicines of the resident, which will include:

- reviewing resident's medication and developing and implementing a pharmaceutical care plan
- assuming prescribing responsibilities
- supporting systematic ordering, prescribing and administration processes with each care home, GP practice and supplying pharmacy where needed
- providing training in care home and GP practice
- communicating with GP practice, care home, supplying community pharmacy and study team

Details of the intervention are in the CHIPPS Service Specification (Appendix 2) developed in previous work packages. It includes the actions, which the PIP will undertake, and are described below.

Reviewing

- review each resident's medication (either face-to-face or based on records)
- ensure clear indication and evidence base for each medication (taking into consideration national and local pathways, guidelines and formularies), informed by tools such as STOPP/START
- minimise the potential for adverse effects
- ensure that the residents receive the most appropriate medication dose
- co-ordinate appropriate monitoring and associated tests for all medicines and conditions
- agree and implement initial care plan with GP, care staff and resident (where appropriate)
- document and maintain records relating to review and develop the care plan in GP and care home records as appropriate

Prescribing

- authorise repeat prescriptions
- co-ordinate appropriate monitoring and associated tests for all medicines and conditions
- change medication doses and formulations
- deprescribe according to agreed pharmaceutical care plan
- document medication changes in GP and care home records and notify supplying pharmacy of all changes to medication within 24 hours
- initiate new medicines only for existing diagnoses or for common ailments which can be managed with medicines classified by the Medicines and Healthcare products Regulatory Agency (MHRA) as Pharmacy (P) or General Sales List (GSL)
- any additional areas of prescribing must be agreed and documented with the GP practice prior to prescribing

Communicating

- agree local protocols for communication with GP practice and care home prior to commencing service. This will include as relevant:
    - Process of communication and messaging
    - The location and expected level of detail of all PIP interventions in the medical records
    - Process and communication of referrals for activities outside the competence of the PIP
- inform supplying community pharmacy about new CHPPS service and role (prior start of service)
    - Communicate all changes in medication to supplying pharmacy
- complete all documentation and recording of activities as required by the study team

Ordering

- support systematic ordering, prescribing, and administration processes with each care home, GP practice and supplying pharmacy
- provide instructions on how to administer each drug
- synchronise resident's prescription quantities for monthly cycles
- add or clarify directions for all medication where it is currently not clear
- provide advice on repeat prescription ordering processes to:
    - minimise missed items
    - optimise quantities
- optimise the use of homely remedies (medicines which can be administered by carers within a defined protocol but without requiring individual authorisation from a prescriber) within the care home
- reconcile resident's medication following a transfer of care

Training provision

- review training needs of care home and GP practice and draft proposed training plan as needed
- provide needs-based training to care home staff
- provide needs-based guidance to relevant GP practice

Safe and effective service provision

- the PIP will be contactable and respond to urgent messages within 24 hours (Monday - Friday)
- the PIP will establish a locally agreed protocol with the GP practice for referral/notification of all medicine related queries from CHIPPS participants to the PIP as appropriate
- the PIP will have full (read/write) access to GP record system to issues prescriptions and update records
- where possible PIP will use remote access to update records when changes are made to GP held record
    - where remote access is not feasible the PIP must update records within 24 hours of making a change
- PIP will have full (read/write) access to care home records to update records during all visits using appropriate local reporting systems
- the PIP will visit/contact the care home once a week on average
- the PIP will visit/contact the GP practice once a week on average
- wherever possible, all annual leave should be agreed before the beginning of the study. In the absence of the PIP (for annual leave, sickness or other reason) a clear system for transfer of responsibility communicated to GP, care home and supplying pharmacy, must also be in place
- the PIP will work within the local prescribing formularies of GP practice and primary care organisation
- the PIP will report and document all significant clinical events including those averted (near misses) using local reporting procedures and study documentation.
- ensure all records are concordant

6.4.1 Compliance and Adherence

The study PIPs will be working closely with the care home staff and the resident's GP, and will be reporting regularly to both parties. The local researcher will also maintain regular contact with the PIP to ensure that study procedures are being properly carried out, according to the study training package. During the study there will be a check of a random 20% sample of the pharmaceutical care plans and associated resident documents by a study geriatrician, to ensure clinical appropriateness and safety. Should any problem arise, the geriatrician will discuss this with the CI and PI. See Section 6.11.2.1 for details.

6.5 Outcomes

Outcome data on residents will be collected at baseline, three months (falls and EQ-5D only) and at the end of the intervention period of six months, using standard, validated approaches:

Primary Outcome

- fall rate per person at 6 months; as documented in care home falls record

Secondary Outcomes

- proxy EQ-5D-5L (quality of life) at baseline, 3 months and 6 months
- face to face self-reported EQ-5D-5L (only applicable for participants with capacity) at baseline, 3 months and 6 months
- proxy Barthel Index (physical functioning) at baseline and 6 months
- fall rate per person in past three months at baseline and 3 months
- health-service utilisation (and associated costs) in past three months at baseline and in past 6 months at 6 months follow up
- mortality
- change in hospitalisation rate per person (baseline rate defined as 3 months prior to randomisation compared with hospitalisation rate at 6 month follow up)
- Drug Burden Index (DBI) at baseline and 6 months

In addition, in the internal pilot stage we will monitor the following to confirm the feasibility of the full RCT:

- sufficient interest from medical practices-PIPs-care home(s) to confirm viability of planned target recruitment numbers and time line
- >30% of eligible patients recruited (from those invited in each home)
- >80% of data available at 3 months for falls data
- no significant intervention related safety concerns

6.5.1 Further Outcomes

6.5.1.2 Process Outcomes

In addition we will collect the following process outcomes per participating resident:

- Number of medications: This will be derived from the medication records, collected for both intervention and control arms, at each stage, and will be used to describe the participant characteristics as well as the intervention (in the intervention group only)
- Use of antipsychotic drugs (a class of medicines used to treat psychosis and other mental and emotional conditions). This will be derived from the medication records collected for both intervention and control arms, at each stage, and will be used to describe the participant characteristics as well as the intervention (in the intervention group only). In addition, this will be included in calculating the DBI in both groups.
- Duplicate drugs: Information about duplicate drugs (both true drug duplicates and therapeutic drug duplication) will be collected in the intervention group by the PIPs when they record this in the PCP as a reason for a PIP intervention and will be used to describe the intervention

6.5.1.3 Health Economic

Costs to the NHS and cost implications for the care home; for full description, see **Section 6.9.5.1,**

For a full list of all the data that will be collected from participants, please see Appendix 3.

6.6 Figure 1. Participant Timeline

			STUDY PERIOD	
	Enrolment	Start of Intervention<br><br>(baseline)		End of intervention
TIMEPOINT	t_0	t_1<br><br>0 months<br><br>(+/- one week)	t_2<br><br>3 months<br><br>(+/- one week)	t_3<br><br>6 months<br><br>(+/- one week)
ENROLMENT:				
Eligibility screen	X			
Informed consent (including a capacity assessment)	X			
INTERVENTION: All participants in the intervention group will receive the intervention which consists of a PIP working with their GP to manage their medicines		→	→	→
DATA COLLECTION				
Qualitative interviews with a purposive sample of residents, relatives, care home staff, GPs and PIPs				x
Proxy Barthel ADL, face to face EQ5D		x	x	x
Proxy EQ-5D-5L		x	x	x
Care home adverse drug events			x	x
Drug Burden Index		x		x
Fall rate per person in past 3 months		x	x	X

Hospitalisations		x		x
Prospective SAE/near miss data recording and notification by PIP, GP and care home staff	Ongoing from t0	Ongoing	ongoing	x
Retrospective SAE/near miss data collection by RA/CTU Trial Manager		X (monthly)	X (monthly)	X

6.6.1 Early Stopping of Follow-up

If the study is stopped prematurely for any reason, the participating PIPs, GP practices, care homes and residents will be informed.

6.6.2 Participant Transfers

If a participant moves from the care home where they have been recruited, they will be withdrawn, because it will not be possible for the intervention to be delivered elsewhere. The data collected until the time at which they withdrew from the study, will be held and used.

6.6.3 Loss to Follow-up

As the care home residents will be living in the participating care home, loss to follow-up is unlikely. If residents are transferred out of the care home then participation will end at date of transfer. Death is not considered as loss to follow-up in this study.

6.6.4 Study Closure

The study ends four months after the end of data collection of last phase of recruitment.

6.7 Randomisation

The study is a 2-arm cluster-randomised controlled trial, which will recruit using recruitment triads. A recruitment triad (i.e. the randomised cluster) consists of 1 GP practice plus 1 PIP plus a mean of 20 residents from at least one care home. Following recruitment of the triad, it will be randomised to either the intervention or control groups. Randomisation will be performed using a web-based electronic randomisation system integrated into the study database.

Figure 2. Study flow chart

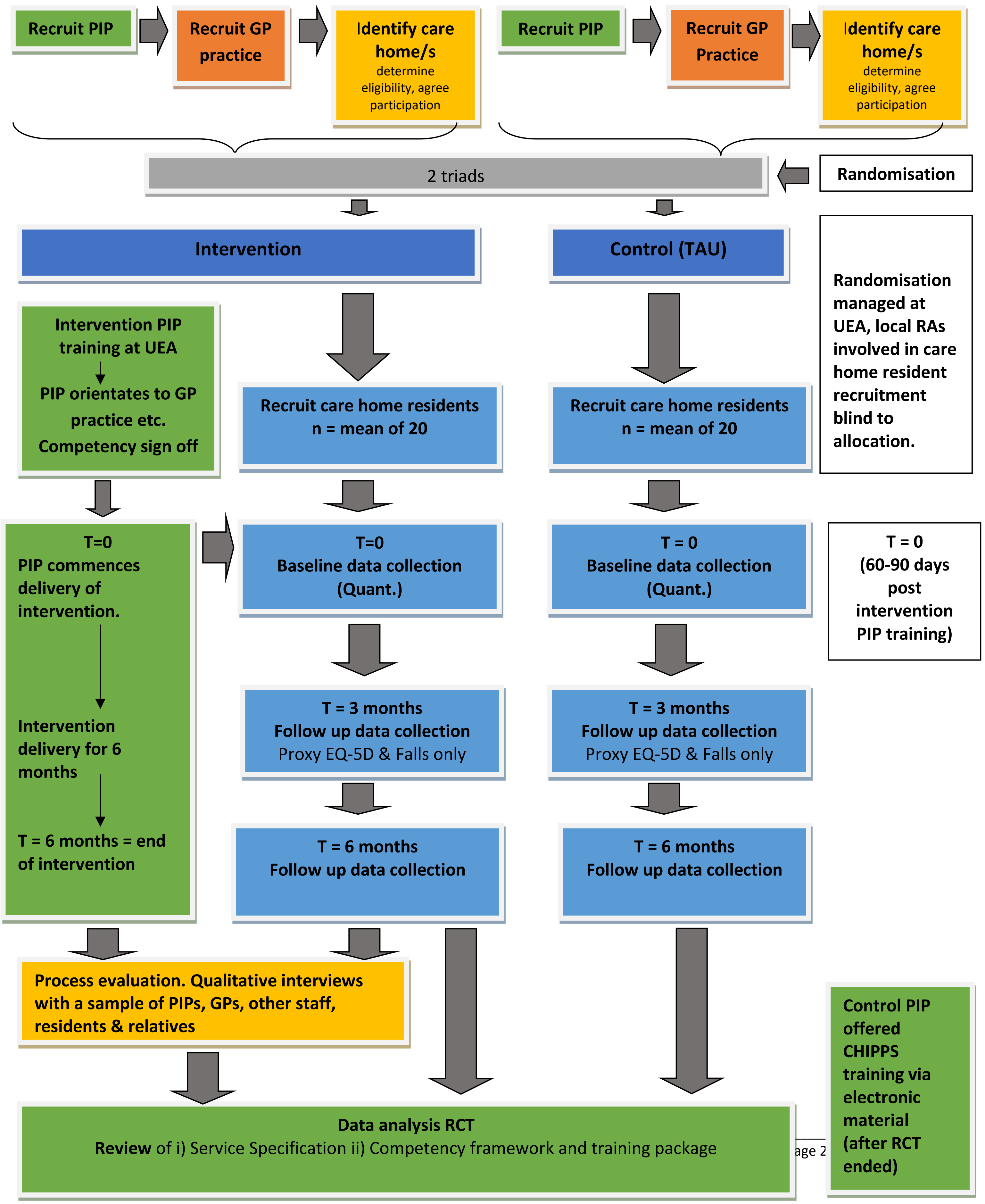

6.8 Sample Size

A sample size of 880 (440 in each arm) would detect a decrease in fall rate from 1.50 per individual over 6 months to 1.178 with 80% Statistical Power. This assumes that the number of clusters available will be 44, with a mean of 20 participants from each, a loss rate of no more than 20% and an ICC of 0.05 or less.

To balance any under recruitment with over recruitment (eventually balancing out over the whole study), we will recruit in all triads a mean of 20 participants. Any variation with these limits would need to be approved by TSG and or DMC.

These assumptions are based upon data from the CAREMED (29) study, which estimated a fall rate of 1.5 per individual over a 6 months period. The detectable difference (from 1.5 to 1.178) is a relative reduction of 21% which is half that detected within a UK based pharmacist led medication review service provided to care homes.

Data from the CAREMED (29) trial indicated a mortality rate of 33% and further loss to follow-up of 5% over 12 months. Thus, a reasonable estimate of total losses due to mortality or other reasons over 6-months would be 20%. However, we will use data, where possible, up to the point at which someone withdrawals from the study (either voluntarily, or otherwise). Therefore, those lost to follow-up, for whatever reason, should contribute some information on falls rate to the study analysis.

6.9 Recruitment and Retention

6.9.1 Recruitment

We will recruit a total of 44 triads; each triad will consist of 1 GP + 1 PIP + a mean of 20 care home residents, from at least 1 care home. Recruitment will be in sequential phases: Pilot, Phase 1, Phase 2 and Phase 3 (see diagram 3 below). Each of these phases encompasses all elements of the study – identification, recruitment and formation of the triads (GP, PIP and care home/s), recruitment of resident participants, randomisation and PIP training for the intervention group.

It is envisaged that recruitment will be equal across all four locations, but some variation across locations is expected.

Figure 3. Pilot and RCT recruitment numbers flow chart by phase and location

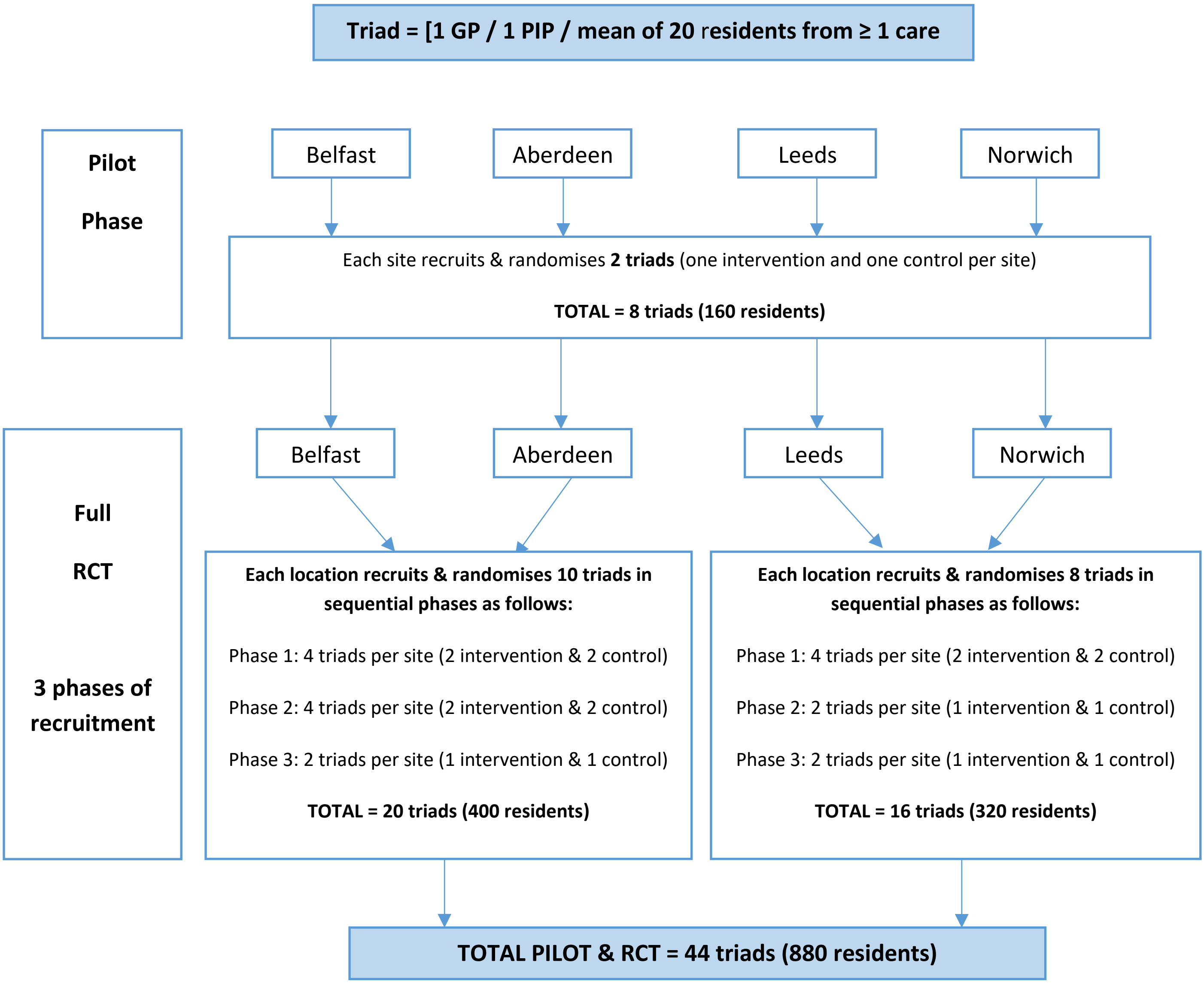

6.9.1.1 PIP recruitment
44 PIPs will be recruited, concurrently with the GP practice with whom they should ideally have an already established close working relationship.

6.9.1.2. GP practice recruitment
Recruitment and consent will be complex due to the need to identify medical practices, recruit homes and then residents.

Ideally, there will be sufficient PIPs expressing an interest in the study, with already established close working relationships with a GP practice. These practices will be approached by local researcher and invited to take part. We will ask for basic demographic information about interested GP practices and their linked care home (e.g. the resident mix and home ownership), so that in the event off excessive interest, we may purposively sample. However, if this does not provide sufficient GP practices, then we will use relevant local research networks and contact medical practices in the area, with a link to care homes, with information about the study and seek expressions of interest.

6.9.1.3 Care home recruitment
The participating GP practice will approach one (or more, if necessary) of their eligible care homes and invite them to take part in the study. Recruitment via the medical practice has been successful in our previous studies, and in the Feasibility Study, and was recommended by care home managers at a PPI meeting. If the care home manager expresses an interest, they will be sent a formal invitation pack by the local researcher (including letter and information sheet). If a care home declines participation, the GP will contact another home and invite them to participate. If there are insufficient residents in one home, then a second home will be recruited.

Where a home does not wish to participate, and there is no alternative home, a different GP practice in that area will be identified and recruited and the process to recruit the care home(s) will be repeated.

6.9.1.4 Resident recruitment
Once we have recruited participating medical practices, PIPs and care homes, GPs will identify, and randomly list, their registered patients resident in the identified care homes taking $\geq$1 medication. The GPs will screen the list against the study inclusion and exclusion criteria and record reasons for exclusion; this data will be collected by the local researcher. The GP practice will send an invitation letter, information sheet (spoken version if necessary) and consent form to each eligible resident in their care, or in the case of residents who the manager knows lacks capacity, to their next of kin. The letters will be sent via the care home managers who will deliver the letters to residents (or in the case of residents who the care home manager knows to lack capacity, post them to their next of kin). The care home manager will visit each resident after at least 24 hours, and obtain verbal consent for the local researcher to be allowed to approach them to discuss participation in the study.

To ensure the recruitment procedure is not biased disproportionately including either those with, or those without, capacity we will ask the care home manager to use the list prepared by the GP and residents will be approached according to the order of the GP list.

The local researcher (see 1.4.3) will then visit the care home and meet with interested residents. They will undertake a brief assessment to determine capacity with residents, using a Capacity Assessment for Residents form, based on a validated tool [5], then obtain fully informed consent from those with capacity, who are willing to participate.

The study will recruit residents both with and without capacity because this is representative of care home populations, where many residents are without capacity, and to exclude such residents would render the research ungeneralizable. In addition, all residents could benefit from the intervention, and so residents without capacity should not be denied the opportunity to participate. For a more detailed justification, please see Appendix 4.

For full details of the procedure for those who lack capacity, taking into account differences in legislation across devolved home nations, please see Section 7.4.

If someone loses capacity during the six months of the study, they will remain in the study. This is one of the points to be agreed in the Consent Form: "I agree to continue participating in the study if I lose capacity before the end of the study". If someone does lose capacity during the course of the study, they will be consented again with a consultee, or WPoA who will be identified in the same manner as described in Section 7.4.

At each follow up visit, the care home manager will be asked if any participants have re-gained capacity. Should anyone regain capacity during the course of the study, we would go through the Patient Information and Consent Form, and if the resident is willing, re-consent them. In Scotland this would be done using the template Resident Recovered Capacity documents, and in England it would be with the original Patient Information and Consent.

Figure **4. Care home resident recruitment flow chart**

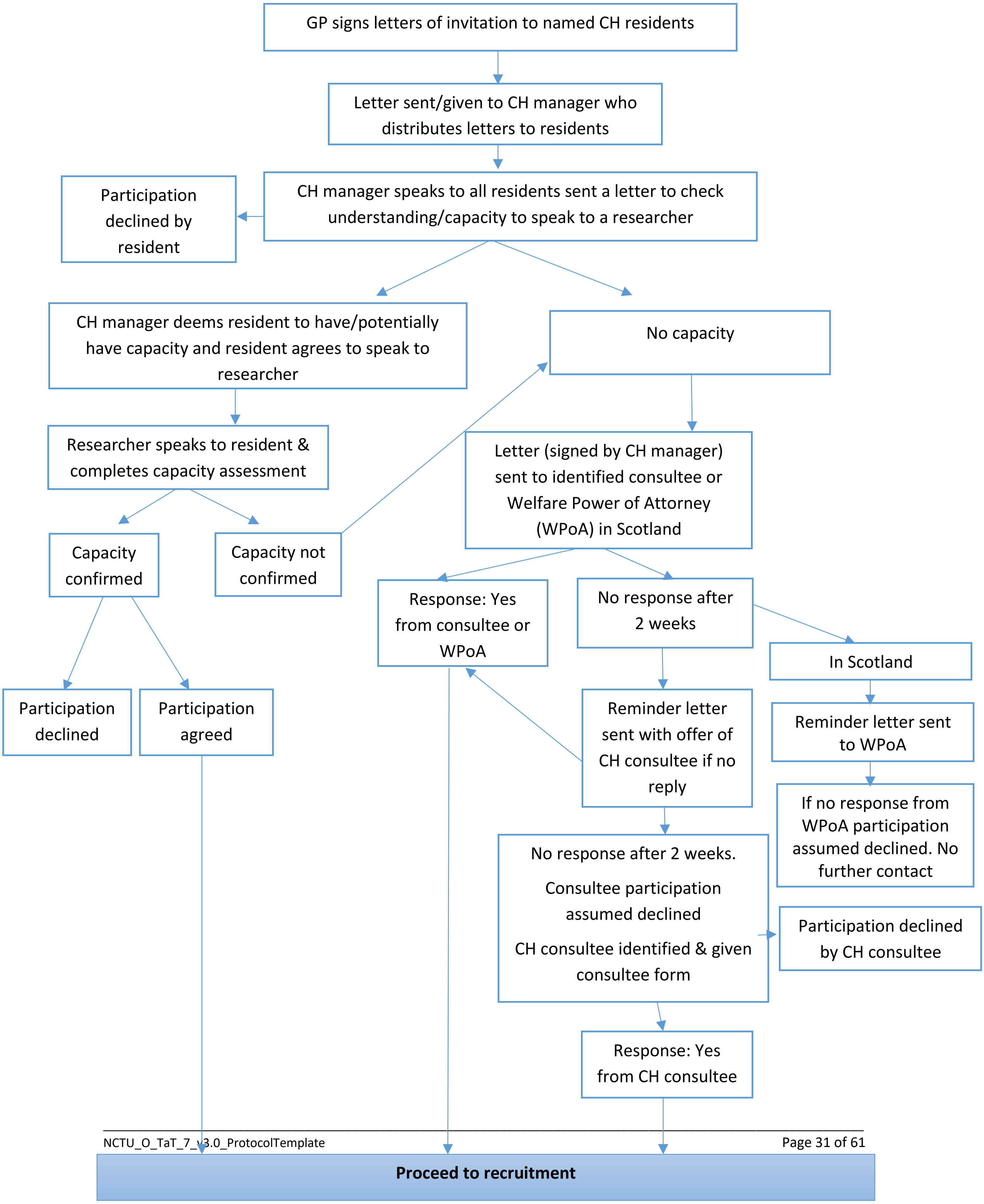

6.9.2 Retention of participants

The intervention will be delivered over a period of six months, during which time we do not expect many participants to drop out. However, it is likely that, due to the age and frailty of the population and based on our experience in previous studies, some of our participants may die in the six months intervention period and some may move. In either case, all data will be collected up to the end of the participant's study participation. However, if a participant dies, or withdraws, before the intervention has begun, then they can be replaced.

If a participant is excluded, as a post randomisation exclusion, they may be replaced, if this can be done within two weeks of the beginning of the intervention.

6.10 Data Collection, Management and Analysis

Data collection, data entry and queries raised by a member of the CHIPPS study team will be conducted in line with the study-specific Data Management Plan, which is held in the e-TMF.

6.10.1 Data Collection Methods

Coded data will be collected, by the local researcher, from the recruitment triad's GP practice and care home record systems, paper and/or digital, as appropriate. Collection of data will be on either paper Case Record Forms (CRFs) or electronically onto tablets.

Identification logs, screening logs and enrolment logs will be kept at each of the four University locations in a locked cabinet within a secured room.

Local research staff will receive study protocol training. All data will be handled in accordance with the Data Protection Act 1998.

6.10.2 Data Management

Data will be entered into the approved centrally held NCTU CHIPPS database by a member of the CHIPPS study team, in each location and protected using established NCTU procedures.

Any data collected with mobile devices may, according to connectivity and interface, either be entered directly to the database or recorded on the tablet in offline mode with synchronisation with the database, in a consistent manner, at a later time.

Coded data: All participants (GPs, care homes and residents) will be given a unique study Participant Identification Number (PIN). Data will be entered under this identification number onto the centrally held database stored on the servers based at NCTU. Access to the database will be controlled with unique usernames and encrypted passwords, and restricted to members of the CHIPPS study team, and external regulators if requested. The servers are protected by firewalls and are patched and maintained according to best practice. The physical location of the servers is protected by CCTV and security door access.

The database and associated code lists have been developed by the Study Coordinators in conjunction with NCTU. The database software (REDCap) provides a number of features to help maintain data quality, including: maintaining an audit trail, allowing custom validations on all data,

allowing users to raise data query requests, and search facilities to identify validation failure/missing data.

After completion of the study the database will be retained on the servers of NCTU for on-going analysis, for 10 years.

The screening and enrolment logs will remain at the care home, or GP practice. For recruitment monitoring purposes, identifiable patient information will be redacted, and pseudoanonymised copies of these logs, then taken to the research office.

Following consent, identifiable (consented participants only) screening data, linking to the Participant Identification Number, will be held locally at the University research office, in a locked filing cabinet. After completion of the study the identification, screening and enrolment logs will be securely archived at each University research office for 10 years unless otherwise advised by NCTU.

6.10.3 Non-Adherence and Non-Retention

Adherence to the service specification by the PIP will be measured in several ways – the PIPs will undertake a CHIPPS training programme which will equip them to deliver the intervention. They will be working closely with the care home staff and the resident's GP, and will report regularly to these parties. The study Senior Programme co-ordinators (see 1.4.4.) will also maintain regular contact with the study PIPs to ensure that study procedures are being properly carried out, according to the study training package.. There will also be a random check of the pharmaceutical care plans and associated resident documents by the study geriatricians, to ensure clinical appropriateness and safety, and a formal process evaluation.

Non-retention of the GPs and/or care homes (due to withdrawal of consent) or care home residents (due to consent withdrawal, death or transfer of care) will be recorded (see also section 6.1.3).

6.10.4 Statistical Methods

6.10.4.1 Statistical Analysis Plan

The primary outcome ('falls per patient') will mostly likely follow a Poisson distribution and a between group comparison to estimate the difference in falls will be made using a Poisson Regression model. This model will include baseline fall rate, prognostic variables (specified prior to analysis) and group as a fixed factor. The unit of analysis will be the individual participant but, due to the study design incorporating 'clustering' these unit outcomes are likely to be correlated. Therefore, a Generalised Estimation Equation (GEE) approach will be used. The Poisson assumption will be assessed with 'fit' statistics and, if appropriate, a Zero Inflated Poisson, or a Poisson model with an over-dispersion term will be considered.

An analogue GEE model will be used for secondary outcomes, with an appropriate change to the error distribution (e.g. Normal).

The estimate of the between group difference will be provided with a 95% confidence interval and tested at the 5% significance level.

A full Statistical Analysis Plan (SAP) will be written prior to the end of data collection and approved by the study DMC.

6.10.4.2 Additional Analyses – Subgroup

There are currently no plans for any subgroup analyses. If this changes, all subgroup analyses will need to be pre-specified in the SAP. An interaction term in the GEE model will most likely be used to estimate subgroup differences in efficacy.

6.10.5 Analysis Population and Missing Data

The primary analysis will be on the 'Intention-to-Treat' population, i.e. all those randomised and analysed according to the arm to which they were randomised.

It is likely that the proportion of missing data will be small. However, if the proportion of individuals with missing falls data is more than 5% a multiple imputation method (using baseline characteristics) will be used as the basis of a secondary sensitivity analysis.

6.10.5.1 Economic evaluations

The objective of the economic evaluation is to estimate the cost-effectiveness of the Pharmacist Independent Prescribers (PIP) intervention.

In line with NICE methods guidance [10] we will estimate costs from the perspective of the NHS and personal social services (PSS). Cost implications for the care home will also be estimated. In order to estimate the levels of resource-use associated with the PIP intervention, building on previous work [8], the PIP will be asked to complete a *PIP activity log*. In addition to patient related activities, the log will request information on 'non-patient specific' activities such as medicines management e.g. reviewing policies/storage of medicines and training other staff. The PIP will also record any patient-related changes e.g. in medication/associated tests in the resident's *Pharmaceutical Care Plan.* Details of the training they receive, including the input from others, will be recorded in their *Personal Development Plan* and from this training costs will be apportioned. Additionally, the use of other NHS and PSS resource-items e.g. district nurse visits services and hospital admissions will be *extracted from care home records* by a local researcher, along with details of the number of pharmaceutical advisory visits undertaken by non-PIP pharmacists. Data in relation to the previous 3 months will be collected at baseline and at 6 months follow-up. Medication details and primary care visit data (GP/practice nurse) will also be extracted from primary care records as it has been shown that there is the 21% which is half that detected within a UK based pharmacist led medication review service provided to care homes use to be under-reported in care home records.

It is possible that the introduction of the PIP could have implications for GP time e.g. GP visits to the care home could be more or less frequent/longer or shorter with a PIP, a medication review/repeat prescriptions may also require less or more GP input. The same may be true for care home staff as medication administration/management in the home may change. It is however unclear that such activities will be recorded in the primary medical notes/care home records. Therefore, in an attempt to try and capture this information, at study registration, GPs will be asked at recruitment to provide information about current activity (frequency, associated time, etc.) in relation to medication reviews, repeat prescriptions and care home visits. Additionally, in staff interviews/focus groups at the end of the study (intervention arm only) GPs will be asked to consider what effect the PIP has

had on such activities i.e. to try and identify whether there are perceptions of associated cost savings (e.g. fewer medication reviews) or cost increases (e.g. more tests/referrals), and the extent of these (if applicable). A similar approach will be taken with care home staff, where this will include asking whether they have done anything in addition to usual practice e.g. training, since the introduction of the PIP. Additionally, we will ask the number of pharmaceutical advisory visits the home has had; this may be, for example, if a Health Board (HB) or CCG pharmacist has visited the care home.

Estimates of resource use will be based on methods used by the applicants previously [9] and will subsequently be attached to items of resource-use in order to estimate the mean overall cost in each study-arm. This will enable the mean incremental cost of the PIP intervention to be estimated.

Quality of life will be assessed via the EQ-5D-5L[6]. For all participants, the proxy version will be completed by a member of the home care staff, such as a nurse or health care assistant who knows the resident; where a participant has capacity a self report version will also be completed. Use of the EQ-5D enables QALY (Quality Adjusted Life Year) scores to be estimated[7]. This will enable the mean difference in QALY scores between usual care (control) and the PIP intervention to be estimated (incremental effect).

The above analyses will enable both the incremental cost and incremental effect associated with the PIP intervention to be estimated. If one option is found to be both less expensive and more effective, then that option would be said to dominate the other and be estimated to be cost-effective. Alternatively, the mean incremental cost effectiveness ratio (ICER) [12] will be estimated, where if this falls below a threshold value of e.g. £20,000 per QALY [7] then that would suggest the more expensive technology was cost-effective. The associated level of uncertainty will also be characterised by estimating cost-effectiveness acceptability curves. Sensitivity analysis will also be undertaken to assess the robustness of conclusions to changes in key assumptions. All analysis will be conducted on an intention-to-treat basis.

6.11 Data Monitoring

6.11.1 Data Monitoring Committee

A Data Monitoring Committee has been established to monitor the trial. Details of membership, relationship with other committees' decision making processes and the timing and frequency of interim analyses are described in detail in the CHIPPS trial DMC Terms of Reference (ToR).

6.11.1.2 Interim Analyses

No efficacy interim analyses are planned. Analysis of recruitment rates, withdrawal rates etc. will be monitored as part of the internal pilot and full trial.

6.11.2 Data Monitoring for Harm

The DMC will be provided with safety data for each treatment arm. This will include frequency of serious adverse events (hospital admissions and deaths,) in both arms and those adverse events which are defined as sudden, unexpected, serious adverse events (likely to be related to the study intervention) in the treatment arm. The committee will advise on the continuation or early stoppage of the trial in the unlikely event that there are concerns over harm to participants.

6.11.2.1 Safety reporting of Serious Adverse Events

The principles of ICH GCP require that investigators and sponsors follow specific procedures when notifying and reporting adverse events or adverse reactions in clinical trials. The processes for the recording of SUSARs, SAEs and AEs and near misses in PIP documentation, GP and care home records, notification to NCTU, CI review, expedited and periodic reporting to REC will be documented in the study specific Safety Management Plan.

For the purposes of this trial, SAEs are defined as in patient hospitalisation and death. The expedited i.e. immediate reporting is required if they are:

- **related** to the study (i.e. they resulted from the intervention) and
- **unexpected**

- referred to hereafter as SUSARs.

A mixture of prospective and retrospective SUSAR notification will be used.

Prospective: GPs will be asked to report SUSARs immediately via a SUSAR Form to a dedicated NCTU safety email address (See section 6.11.2.1.2). GPs should report any SUSARs related to the PIP intervention from the beginning of the intervention until 30 days after the intervention.

Retrospective: A systematic retrospective collection of SAEs will be conducted in both intervention and control practices, whereby the NCTU Trial Manager will contact every participating care home once a month and ask about any SAEs.

Deaths and hospitalisations in both arms will also be reported to REC via the annual report.

6.11.2.1.1 Causality

The causality assessment of the SAE should be given by the GP. If the GP identifies a positive causality (i.e. the SAE is linked to the PIP intervention and is therefore a SUSAR) then this is signed off by the CI. The GP must assess the causality of all serious adverse events in relation to the PIP intervention using the definitions in the table below. If the event is classified as 'serious' and assessed as being related to the PIP intervention then an SUSAR form must be completed and NCTU notified within 24 hours (see 6.11.2.1.2. below)

Table 1: SAE Causality Definitions

**Event type**	**Causality assessment**	**Description**
SAE	Unrelated	There is no evidence or rationale for any causal relationship.
SUSAR	Likely to be related	There is evidence, and a rationale, to suggest a causal relationship and other possible

		contributing factors can be ruled out.

6.11.2.1.2 Notification of SUSARs and Safety Concerns to the NCTU

NCTU must be notified of all SUSARs within 24 hours of the GP becoming aware of the event.

The SUSARE form must be emailed to the NCTU SAE reporting email address: nctu.safety@uea.ac.uk

All staff involved in the care of study participants (i.e. PIPs, care home staff, any other health care professionals) will also be asked to report immediately, to a separate dedicated email address (chipps.safety@uea.ac.uk), any events about which they are concerned.

NCTU can be prospectively notified of any further safety concerns or near misses by all staff involved in the care of study participants via a study specific safety email address: Chipps.safety@uea.ac.uk

6.11.2.1.3 Notification of SUSARs and Safety Concerns to the DMC

The independent DMC will review the frequency of SAEs (deaths and hospitalisations) and falls. The form and frequency of DMC review of safety data will be documented in the Safety Management Plan and the DMC Terms of Reference (both documents are held in the e-TMF). In addition, further monitoring and substantiation of the process will be undertaken at the recommendation of the DMC.

The DMC will review unblinded data to evaluate safety measures at GP practice level (i.e. across all care homes associated with the GP practice) and at care home level (i.e. within each individual care home).The DMC will review safety data on a regular basis and recommend actions to the PSC and via the PSC to the Trial Management Group. If safety is identified as an issue by any of the trial oversight groups a substantial amendment to the protocol would be submitted to REC.

6.11.3 Quality Assurance and Control

The frequency, type and intensity of routine and triggered on-site monitoring is detailed in the CHIPPS Quality Management and Monitoring Plan (QMMP).

6.11.3.1 Risk Assessment

The Quality Assurance (QA) and Quality Control (QC) considerations for the CHIPPS study are based on the standard NCTU quality management processes that includes a formal Risk Assessment, and that acknowledges the risks associated with the conduct of the study and proposals of how to mitigate them through appropriate QA and QC processes. Risks are defined in terms of their impact on: the rights and safety of participants; project concept including study design, reliability of results and institutional risk; project management; and other considerations.

QA is defined as all the planned and systematic actions established to ensure the study is performed and data generated, documented and/or recorded and reported in compliance with the principles of GCP and applicable regulatory requirements. QC is defined as the operational techniques and activities performed within the QA system to verify that the requirements for quality of the study related activities are fulfilled.

6.11.3.2 Monitoring of pharmaceutical care plans (PCPs)

To quality assure the interventions delivered by the PIPS a random sample of 20% of the PCPs will be reviewed for clinical appropriateness and identification of any safety concerns by one of the study geriatricians (Section 7.4.1). The selection of PCPs for review will be based on the following for each PIP:

- review random 2 out of first 5 PCPs (as they are highest risk after previous weeks)
- review random 1 out of next 5 PCPs (unless low level concerns, in which case 2 out of 5, moderate level concerns 3 out of 5, significant concerns after first five = Stop)
- review random 1 out of next 5 PCPs (unless low level concerns remain – in which case 2 out of 5, if moderate level concerns remain consider Stopping at half-way point)
- review 0 out of last 5 PCPs (assuming no concerns in previous, unless low level concerns remain in which case one should be sampled)

6.11.3.3 Central Monitoring at NCTU

NCTU staff will review electronic Case Report Form (CRF) data for errors and missing key data points. The study database will also be programmed to generate reports on errors and error rates. Essential study issues, events and outputs, including defined key data points, will be detailed in the CHIPPS study Data Management Plan.

6.11.3.4 On-site Monitoring

The frequency, type and intensity of routine and triggered on-site monitoring will be detailed in the CHIPPS Quality Management and Monitoring Plan (QMMP). The QMMP will also detail the procedures for review and sign-off of monitoring reports. In the event of a request for a study location inspection by any regulatory authority, NCTU must be notified as soon as possible.

6.11.3.4.1 Direct access to participant records

Participating investigators will agree to allow study related monitoring, including audits, REC review and regulatory inspections, by providing access to study related documentation as required. Participant consent for this will be obtained as part of the informed consent process for the study.

6.11.3.5 Study Oversight

Study oversight is intended to preserve the integrity of the study by independently verifying a variety of processes and prompting corrective action where necessary. The processes reviewed relate to participant enrolment, consent, eligibility, and allocation to study groups; adherence to study interventions and policies to protect participants, including reporting of harms; completeness, accuracy and timeliness of data collection; and will verify adherence to applicable policies detailed in the Compliance section of this protocol (Section 6.4.1). Independent study oversight complies with the NCTU trial oversight policy.

This oversight applies both overall and for each location by reviewing the study dataset or performing site visits as described in the CHIPPS Quality Management and Monitoring Plan.

6.11.3.5.1 Trial Management Group

As this trial is one of the work packages in the research programme, the Programme Management Group (PMG), will assume the role of Trial Management Group and will oversee the trial

6.11.3.5.2 Programme Steering Committee

The Independent Programme Steering Committee Steering Committee (PSC) is the independent group responsible for oversight of the study in order to safeguard the interests of study participants. The PSC provides advice to the CI, NCTU, the funder and sponsor on all aspects of the study through its independent Chair. The membership, frequency of meetings, activity (including study conduct and data review) and authority will be covered in the PSC terms of reference.

6.11.3.5.3 Study Sponsor

The role of the sponsor is to take on responsibility for securing the arrangements to initiate, manage and finance the study. When an institution is the Study Sponsor and has delegated some and/or the totality of Sponsor's responsibilities to the NCTU, the Sponsor's form for delegated responsibilities should be completed and signed by all parties before the start of the study.

7 Ethics and Dissemination

7.1 Research Ethics Approval

Before initiation of the study at any care home, the protocol, all informed consent forms and any material to be given to the prospective participant will be submitted to HRA and the relevant RECs for approval. Any subsequent amendments to these documents will be submitted for further approval prior to use. Before initiation of the study at each additional local research office centre the same/amended documents will be submitted to local Research and Development (R&D) for information.

The rights of the participant to refuse to participate in the study without giving a reason will be respected. The participant remains free to change their mind at any time about the protocol treatment and follow-up without giving a reason and without prejudicing their further treatment.

7.2 Other Approvals

The protocol will be submitted for formal approval and methodological, statistical, clinical and operational input from the NCTU Protocol Review Committee prior to submitting for any external approvals.

In addition to submitting to HRA/REC (and the Scottish REC A), the protocol and all relevant supporting documentation, will also be submitted for information to local R&D committees.

7.3 Protocol Amendments

Any substantial protocol amendments will be agreed by the Trial Management Group. Then the appropriate documents will be completed, including an explanation for the amendment, and the appropriate bodies informed – Ethics Committee, HRA, NHS R&D, funder and sponsor. If the substantial amendment impacts on delivery of the intervention, the appropriate persons will also be informed – PIPs, participating GP practices, care homes and, if appropriate participants.

7.4 Consent or Assent (in the case of residents without capacity)

Participating GPs will identify care home residents meeting the inclusion criteria, and send an invitation letter, information sheet (spoken version if necessary) and consent form to each eligible resident in their care, or in the case of residents who lack capacity, to their Consultee or WPoA. The GP will ask the care home managers to deliver the letters to residents (or in the case of residents who lack capacity, post them to their Consultee or WPoA), and then visit each resident after at least 24 hours, and ask if the resident is happy to talk to the local researcher about the study. If the care home manager thinks that the resident does not have sufficient capacity to agree to consent to a visit from the local researcher, then a consultee or WPoA will be identified (method described below). If the resident indicates that they would prefer to discuss this with a relative/friend present, the care home manager and local researcher will seek to accommodate this.

The local researcher will then visit the care home and all willing residents, and go through what is involved in participating in the study. The researchers will also administer the Capacity Assessment for Residents form (for which they will have received training) to establish capacity. If it is clear after the initial introduction of the research to the resident that a resident lacks capacity, the assessment tool will not be administered and the resident's consultee/WPoA will be contacted. If the resident is found to have capacity, and is willing, they will undergo informed consent and be recruited.

Where a resident lacks capacity either to consent to a visit from the researcher, as identified by the care home manager or to undertake fully informed consent to participate in the study, as identified by the researcher, a consultee/Welfare PoA will be identified and approved as below.

Different approaches will be taken in Scotland and England and Northern Ireland.

In England and Northern Ireland, a letter will be sent to their supporting relative/friend/potential consultee from the GP, enclosing the Information Sheet and Advice Form for signature. The Information Sheet explains the study and asks if, in their opinion, their friend/relative would have wanted to participate, if they had been able to decide this for themselves. If they felt that their friend /relative would have participated, the letter asks if they would they be willing to act as consultee and give permission on their friend/relative's behalf? The letter asks the potential consultee to complete this Advice Form and send it back within two weeks. If there is no response within two weeks, another similar letter is sent, asking for return of the completed Advice Form within one week, stating that, if this is not returned the care home will assume that the friend/relative cannot be a consultee and will then identify someone for this role from within the care home; this person will not be the care home manager and will be completely independent of the study.

In Scotland, the letter requesting permission, on behalf of the resident, will be sent to the resident's Welfare PoA, along with Information Sheet and Consent Form in the approved Scottish template. If there is no response within two weeks, another similar letter is sent. If the Welfare PoA returns the signed consent form, then the resident will be recruited onto the study. If the Welfare PoA does not return the form, the resident will not be recruited.

In England, capacity to consent is described in the Mental Capacity Act 2005 and Department of Health Guidance for nominated consultees (OPSI 2005, DH 2008) and involves using personal and nominated consultees. The assent process is consistent with Alzheimer Europe Ethics of Dementia Research (http://www.alzheimer-europe.org/Ethics/Ethical-issues-in-practice/Ethics-of-dementia-research/Informed-consent-to-dementia-research) and has the support of Alzheimer's UK.

In Scotland, capacity to consent is described in the Adults with Incapacity Act (Scotland) 2000 http://www.legislation.gov.uk/asp/2000/4/section/16, and involves a WPoA who is able to give consent.

In Northern Ireland there is currently no primary legislation on capacity (according to the General Medical Council) and so decisions about medical treatment and care when people lack capacity must be made in accordance with the common law, which requires decisions to be made in a person's best interests. In Northern Ireland we will follow the procedures used in England.

The provision of informed consent to participate in the study includes consenting to data collection and to participating in study follow up as described in the protocol and the participant information sheet. Participants will be requested to consent to their medical records, including GP records, being accessed as part of the study and for supplementary data to be collected on their health service use.

The Principal Investigator (PI) at each University retains overall responsibility for the informed consent of participants at their site and will ensure that any person delegated responsibility to participate in the informed consent process is duly authorised, trained and competent to participate according to the ethically approved protocol, principles of Good Clinical Practice (GCP) and Declaration of Helsinki.

The right of a participant or consultee/WPoA to refuse participation without giving reasons will always be respected. The participant and their consultee/WPoA(S) as appropriate will remain free to withdraw from the study at any time without giving reasons and without prejudicing his/her further treatment and will be provided with a contact point where he/she may obtain further information about the study. The PI takes responsibility for ensuring that all vulnerable participants are protected and participate voluntarily in an environment free from coercion or undue influence.

Informed consent will be collected from each participant before any data, related to the study, are collected. The original consent form will be kept in the Site File at the Research Site and four copies will be taken. One copy of this will be kept by the participant, one will be retained in the resident's care home file, a third will go to Norwich Clinical Trials Unit for monitoring and the fourth will be retained at the GP practice. The Norwich Clinical Trials Unit copy is only stored temporarily, for monitoring purposes and will deleted once reviewed. This copy is sent through uploading onto the REDCap database and is securely held in a section which is separate from data; access will be restricted to only those users who need it, such as researchers. Other users, for example data analysts, statisticians, will not have access to the consent forms, only the pseudoanonymised data.

Consent, and Assent where appropriate, will be recorded in the resident care home notes.

Should there be any subsequent amendment to the final protocol, which might affect a participant's participation in the study, continuing consent will be obtained using an amended Consent form which will be signed by the participant (or consultee as appropriate).

7.4.1 Process Evaluation

At the end of the intervention, semi-structured in-depth interviews will be undertaken by University researchers at each location with a purposive sample of three of each of the following: GPs, care home managers, staff, residents and relatives (if available). The interviews will determine views of

the PIP service and depending on the perspective of the interviewee the interviews will consider service implementation and delivery, perceived effectiveness of the intervention and the identification of any unintentional consequences. Barriers and facilitators to delivering the service will be identified.

All stakeholders interviewed will be given an information sheet and consent form prior to participation in the interviews. All interviews will be audio recorded and transcribed verbatim, by an experienced transcriber at UEA and thematically analysed to identify themes emerging inductively from the data.

A random sample of pseudoanonymised care plans will be obtained from each PIP and reviewed by a study geriatrician in the Programme Management Group (Section 6.11.2.1). This information will be used to assess intervention fidelity and safety/appropriateness of decision making and will be utilised within the process evaluation to describe PIP activity. The PIP Activity Log, associated with the trial for economic evaluation, will also further facilitate intervention fidelity checking.

Focus groups will be held with PIPs at each site to review both the process and the experience of delivering the intervention and the training package.

7.5 Confidentiality

All participants will be assigned a unique identification number which will be used on all data generated, consequently all data held will be pseudoanonymised. All paper based records with participant information will be stored in locked filing cabinets in locked offices. Lists of participants and their identification numbers will be stored in a different locked cabinet, separate from the data and only members of the study research team will have access to these cabinets.

Data will be collected on paper, or electronically onto tablets, and will be entered onto a secure, password protected Web-based database and held pseudo anonymously. Participant details will be stored electronically in a different secure, password protected Web-based database, with only the research team having the ability to access both data and details if required. The secure Web-based database will be held on UEA's secure servers and appropriately backed-up.

The local researchers will collect data from participating GP practices and care homes – if this is collected on paper, the researchers will be trained on safe transport of the paper back to the secure local research site office. If data are collected electronically, the data will be held on a tablet or an encrypted data stick while being transported back to the secure local research site office.

If data are being sent to sponsors or co-investigators, they will always be pseudo anonymous and contain no identifying participant details; in addition they will be encrypted using a zip file.

7.6 Declaration of Interests

The investigators named on the protocol have no financial or other competing interests that impact on their responsibilities towards the scientific value or potential publishing activities associated with the study.

7.7 Indemnity

PIPs

PIPs participating in the study will hold professional indemnity insurance cover for undertaking their normal work and also work as part of a study in a care home, for the duration of the study. GP practices selected to participate in this study will provide clinical negligence insurance cover for harm caused by their employees and a copy of the relevant insurance policy or summary shall be provided to UEA, upon request.

Sponsor

As study sponsor, NHS South Norfolk CCG have appropriate indemnity to cover their responsibilities as Sponsor and any liability in respect of this.

UEA

UEA holds insurance to cover participants for injury caused by their participation in the study Participants may be able to claim compensation if they can prove that UEA has been negligent. However, as this study is being carried out in a care home, the care home continues to have a duty of care to the participant in the study; UEA does not accept liability for any breach in the care home's duty of care, or any negligence on the part of care home employees. This does not affect the participant's right to seek compensation via the non-negligence route.

7.8 Finance

CHIPPS is fully funded by National Institute for Health Research (NIHR) grant number [RP-PG-0613-20007].

7.9 Archiving

The research team at UEA agree to archive and/or arrange for secure storage of CHIPPS study materials and records for a minimum of 10 years after the close of the study unless otherwise advised by the NCTU.

7.10 Access to Data

Requests for access to study data will be considered, and approved in writing where appropriate, after formal application to the TMG/PSC. Considerations for approving access are documented in the PMG/PSC Terms of Reference.

7.11 Ancillary and Post-study Care

Nothing planned

7.12 Publication Policy

7.12.1 Study Results

The results of the study will be disseminated, regardless of the direction of effect, in accordance with the publication policy as agreed by the Programme Management Group and will include presenting the study at conferences, professional briefings and as feedback to participants.

7.12.2 Authorship

Authorship will be according to the publication policy as agreed by the Programme Management Group.

7.12.3 Reproducible Research

The trial protocol will be published and made available for public access throughout the trial period.

8 Ancillary Studies

None

9 Protocol Amendments

None

10 References

1. Chan AW, Tetzlaff JM, Altman DG et al. SPIRIT 2013 Statement: Defining Protocol Items for Clinical Trials. Ann Intern Med 2013; 158:200-207.

2. Chan AW, Tetzlaff JM, Gotzsche et al. SPIRIT 2013 explanation and elaboration: guidance for protocols of clinical trials. BMJ 2013; 346: e7586.

3. Alldred DP, Kennedy M-C, Hughes C, Chen T, Miller P Interventions to Optimise prescribing for Older People in Care Homes. Cochrane Database of Systematic Reviews 2016, Issue 2. Art. No.: CD009095. DOI: 10.1002/14651858.CD009095.pub3.

4. Alldred DP, Raynor DK, Hughes C et al. (3 more authors) (2013) Interventions to optimise prescribing for older people in care homes. Cochrane Database of Systematic Reviews, Issue (2). CD009095. ISSN 1469-493X

5. Siervo M, Bunn D, Prado CM, and Hooper L. AJCN 2014. Accuracy of serum osmolarity in older people. Online Supplementary Text Files, 24

6. Herdman M, Gudex C, Lloyd A, Janssen M, Kind P, Parkin D, Bonsel G, and Badia X Development and preliminary testing of the new five-level version of EQ-5D (EQ-5D-5L). Qual Life Res 2011; 20: 1727-1736.

7. Sculpher M, Drummond M, and Buxton M The iterative use of economic evaluation as part of the process of health technology assessment. J Health Serv Res Policy 1997; 2: 26-30.

8. Curtis L and Burns A, Unit Costs of Health and Social Care 2015. University of Kent, 2015.

9. Sach T, Desborough J, Houghton J, and Holland R Resource Use Measurement In Trials Conducted In Care Homes: A Study Of Level-Of-Agreement Between Data Collected From GP Records And Care Home Records. Value in Health 2015; 18: A689.

10. NICE Guide to the methods of technology appraisal 2013. National Institute of Health and Clinical Excellence (NICE) publications, 2013.

11. Zermansky AG, Alldred DP, Petty DR, Raynor DK, Freemantle N, Eastaugh J and Bowie P; Clinical medication review by a pharmacist, Issue 6, 1 November 2006; 586–591, https://doi.org/10.1093/ageing/afl075

    https://academic.oup.com/ageing/article/35/6/586/14651

11 Appendices

Appendix 1. The PIP - further information for CHIPPS

The Pharmacist Independent Prescriber (PIP)

Following a four-year undergraduate programme, a one year postgraduate training year and a successful outcome at the registration exam, a pharmacist can register with the General Pharmaceutical Council (GPHC). They must work in pharmacy practice for two years before they become eligible to participate in further training which will qualify them as a Pharmacist Independent Prescriber.

The regulations that allow a pharmacist to prescribe independently came into effect in 2006. A pharmacist can only prescribe independently following successful completion of a GPHC accredited independent prescribing (IP) course and once they have applied and been approved for annotation as an IP on the GPHC register.

The GPHC accredited courses are delivered by universities and comprise of a part-time distance-learning programme with a residential face-to-face period, during which time the pharmacists are assessed (minimum of 26 days of teaching and learning). They then produce evidence from a 12 day period of working in practice which must be signed off by their designated medical prescriber mentor. The university exam board then ratifies the assessment marks and awards the IP qualification to those that have passed.

Pharmacists routinely carry out medication reviews and make recommendations to the patient's doctor regarding clinical and cost effective treatment for their patients. As an independent prescriber a pharmacist can prescribe and de-prescribe autonomously for any patient with any condition within their clinical competence. Thus, with the IP qualification a pharmacist can assume responsibility for the patient's medication. The PIP however, does still communicate with both the patient and the doctor regarding any changes. There are several reasons why a patient's medication may need to be reviewed: adverse drug reactions, side-effects, inappropriate or ineffective therapy, and/or redundant therapy.

In this research project the pharmacists, who will have already successfully completed their Pharmacy Independent Prescribing course, will receive additional training specifically related to the tasks they will be required to undertake for the study. This training will target specific therapeutic areas that the PIP is likely to encounter in the care homes; e.g. anticoagulation, benzodiazepines, antipsychotics, patients with dementia, Parkinson's etc. The role of the PIP will be to ensure they maximize the therapeutic benefit of the patient's medication while minimizing the risk to the patient e.g. falls risk etc.

Appendix 2. Service Specification

CHIPPS Care Homes Independent Pharmacist Prescribing Study

Care Homes Independent Pharmacist Prescribing Study (CHIPPS)

Service Specification

1. Service outline

CHIPPS is a National Institute for Health Research (NIHR) programme grant to develop and deliver a cluster randomised controlled trial to determine the effectiveness and cost-effectiveness of making pharmacist prescribers part of a team working alongside care home staff and General Practitioners (GPs) in care homes for older people. CHIPPS will provide a Pharmacist Independent Prescriber (PIP) to review and optimise prescribing in recruited residents and facilitate and support cost effective evidence based prescribing and medicines management in care homes for older people.

2. Aims and objectives

The aim of the service is to improve health outcomes and wellbeing of care home residents and ensure medicines are prescribed and managed in a safe, effective and cost effective way.

In order to meet the stated aims recruited GP practices and care homes will work with a PIP who has demonstrated competency in care home medicines management and prescribing in older people. The PIP will be based at the GP practice, for the duration of the study, and will have developed an excellent working relationship with the GP practice and care home prior to commencing the service delivery. The service will run for a period of 6 months.

3. Inclusion/exclusion criteria for service

3.1 Pharmacist Independent Prescriber (PIP)

Inclusion criteria

- Registered as a pharmacist independent prescriber
- Following training can demonstrate competence to deliver service (See section 4)
- Ability to work flexibly and commit a minimum of 16 hours a month to deliver the service for three months

Exclusion criteria

- Substantive employment with the community pharmacy (branch/store) which supplies medicines to the care home with which the PIP would work

3.2 Care home

Inclusion criteria

- Care Quality Commission (CQC) registered specialism as caring for adults over 65
- Primarily caring for residents over 65 years

Exclusion criteria

- Care homes who receive additional medication focussed services with a visit frequency ≥ monthly
- Care homes which only provide carer or support remotely (They do not have carers on site 24 hours a day)
- Care homes which are currently under formal investigation with the Care Quality Commission (CQC) or equivalent body

3.3 Residents

Inclusion criteria

- Resident under the care of the participating GP practice
- Residents currently prescribed at least 1 medicine
- Residents or their appropriate representative who are/is able to provide informed consent/assent
- Permanent resident in care home (not registered for respite care/temporary resident)
- Residents must be 65 years or over

Exclusion criteria

- Residents who are currently receiving end of life care (equivalent to yellow (stage C) of the Gold Standards Framework prognostic indicator)
- Resident with additional limitations on their residence (e.g. held securely)
- Participating in another research study

4. Service requirements

4.1. Recruitment and employment of the Pharmacist Independent Prescriber (PIP)

- Initial identification and recruitment of the PIP will be conducted by the CHIPPS management committee
    - The PIP will require:
        - Excellent interpersonal, communication and IT skills
        - Familiarity with relevant GP software systems
        - Experience of providing prescribing and medicines management advice and support
        - Previous experience of working in GP practice environment

- Be able to travel to site locations
- A mobile phone to be contactable for the purposes of delivering this service
- Appropriate indemnity insurance for prescribing

- PIP will be employed according to local arrangements and seconded to the relevant GP practice for the duration of the study and during training and competency assessment (see section 4.2)

4.2 Training and competency assessment of PIP

See Appendix 1

4.3 PIP roles and responsibilities [NB: categorised as essential or not]

The PIP will, where appropriate:

4.3.1 Review each resident's medication and develop and implement a pharmaceutical care plan[2] (essential)

- Optimise prescribing ensuring clear indication and evidence base for each medication (taking into consideration national and local pathways, guidelines and formularies), informed by tools such as STOPP/START
- Minimise the potential for adverse effects
- Optimise the dose of all medication
- Co-ordinate appropriate monitoring and associated tests for all medicines and conditions
- Agree initial care plan with GP, care staff and resident (where appropriate)
- Document and maintain records relating to review and care plan in GP and care home records as appropriate

4.3.2 Prescribing (essential)

- Authorise repeat prescriptions
- Co-ordinate appropriate monitoring and associated tests for all medicines and conditions
- Deprescribe medicines according to agreed pharmaceutical care plan
- Document medication changes in GP and care home records and notify supplying pharmacy of all changes to medication within 24 hours
- Only initiate new medicines for existing diagnoses or for common ailments which can be managed with medicines classified by the Medicines and Healthcare products Regulatory Agency (MHRA) as Pharmacy (P) or General Sales List (GSL)
- Any additional areas of prescribing must be agreed and documented with the GP practice prior to prescribing (e.g. antibiotics for simple UTIs)

---

[2] A Pharmaceutical care plan is defined as plan for the responsible provision of medicine-related care for the purpose of achieving defined outcomes that improve the patient's quality of life. It involves gathering information, identifying problems, assessing problems and desired improvements.

4.3.3 Communication (essential)

- Agree local protocols for communication with GP practice and care home prior to commencing service. This should include:
  - Process of communication and messaging
  - The location and expected level of detail of all PIP interventions in the medical records Process and communication of referrals for activities outside the competence of the PIP
- Inform supplying community pharmacy about service and role (prior start of service)
  - Communicate all changes in medication to supplying pharmacy
- Complete all documentation and recording of activities as required by the study team

4.3.4 Support systematic ordering, prescribing, and administration processes with each care home, GP practice and supplying pharmacy where needed: (undertaken at PIP's discretion)

- Provide instructions on how to administer each drug
- Synchronise residents prescription quantities for monthly cycles
- Add or clarify directions for all medication where it is currently not clear
- Provide advice on repeat prescription ordering processes to:
  - Minimising missed items
  - Optimising quantities
- Optimise the use of homely remedies within the care home
- Reconcile resident medication following a transfer of care

4.3.5 Training provision (undertaken at PIP's discretion)

- Review training needs of care home and GP practice and draft proposed training plan
- Provide training to care home staff on training needs basis from agreed list of potential topics/areas
- Provide guidance to relevant GP practice on training needs basis from agreed list of potential topics/areas

4.4 Safe and effective service provision

- PIP will be contactable and respond to messages within 24 hours (Monday - Friday)
- The PIP will establish a locally agreed protocol with the GP practice for referral/notification of all medicine related queries from CHIPPS participants to the PIP as appropriate (see 4.3.5)
- PIP will have full (read/write) access to GP record system to issues prescriptions and update records
- Where possible PIP will use remote access to update records when changes are made to GP held record
  - Where remote access is not feasible the PIP must update records within 24 hours of making a change

- PIP will have full (read/write) access to care home records to update records during all visits using appropriate local reporting systems
- The PIP will visit/contact the care home at least once a week
- The PIP will visit/contact the GP practice at least once a week
- Wherever possible, all annual leave should be agreed before the beginning of the study. A clear system for transfer of responsibility communicated to GP, care home and supplying pharmacy
- The PIP will work within the local prescribing formularies of GP practice and primary care organisation.
- The PIP will report and document all significant clinical events or near misses using local reporting procedures and study documentation.
- Ensure all records are aligned

5. Outcomes from service

As part of the feasibility study, we will measure levels of resource-use associated with the PIP intervention, which will be estimated using the PIP log (Appendix 2), which the PIP will complete every day.

6. End of service transitional arrangements

The duration of service will be clearly documented in study documentation and signed agreement to service provision completed by Care Home and GP practice prior to commencing the study.

- All original policy and procedure documentation will be kept prior to amendments made during service provision
- Transfer meeting with PIP, GP practice and care home at least 3 weeks prior to end of service
    - Agree transfer of responsibilities from PIP
        - Agree named contact point for medication issues at GP practice
    - Communicate current plans for each resident
        - Transfer of care plan and set review date
    - Agree changes in policy and procedures

Appendix 3. List of data accessed from residents' records

Data will be extracted using the following documents:

1. Pharmaceutical Care Plan (completed and used by PIPs for the purpose of undertaking medicines review)
2. Resource use questionnaire (to be extracted from primary care records for health economic purposes)
3. Resource use questionnaire (to be extracted from care home records for health economic purposes)

1. Pharmaceutical Care Plan

For PIP use only

- Resident Name
- Date of birth
- Gender
- Key contact/relative
- Other contact numbers
- Care home
- Resident classification in care home
- Date of entry to care home

Accessible to researcher

- Participant study ID
- PIP ID
- Residents age at point of recruitment
- Medical history (Active problems)
- Medical history (Significant Past Problems
- Known allergies (specify or none known)
- Self- medicating (Y/N)
- Medicine administered covertly (Y/N)
- Nutritional support (Y/N)
- Incontinence of urine (Y/N)
- Incontinence of faeces (Y/N)

- Mobility (Immobile, walk with aids/walk unaided)
- Fall risk (Y/N)
- Data Sources used e.g. GP records, NHS Kardex, Homely remedies, falls book or other
- PIP initial review date
- Medicines reconciliation
    - Form
    - Strength
    - dose
    - Frequency
    - Duration
    - Date of initial Review
    - Suggested intervention
    - Outcome & action (date)
- OTC/herbal medication/homely remedies used (Y/N) specify
    -
- Tests:
    - Date of test
    - Heart rate
    - BP
    - Renal function
    - Liver function
    - Cholesterol
    - Hb
    - MCV
    - Blood sugar
    - Height
    - Weight
    - BMI
    - Other tests

2. Resource use questionnaire (to be extracted from primary care records)

- Participant study ID
- Baseline date/Follow up date

- Contacts with a GP / Practice nurse and other HCPs including Outpatients in the past three months
    - Face to face contact
    - Telephone contact
- In the past 3/6 months has the participant stayed overnight in a hospital or another healthcare facility for any reason?
    - No
    - Yes – details of number of nights spent/type of unit/if an emergency admission
- Medications prescribed in past three/six months: Tablets/capsules/injections/implants/patches/liquids
    - Name of medication
    - Pack size and units
    - Has pt been on med more than 12 months
    - Date started
    - Dose
    - Units
    - Frequency
    - Date stopped
    - Number of packs prescribed (in the last three months)
    - On MAR chart at start of intervention/follow up date?
- Tubs/tubes/bottles/creams/inhalers/sprays/drops (excluding dressings)
    - Name of medication
    - Date started
    - Number of prescriptions issued(in the last three/six months)
    - Date stopped
    - On MAR chart at start of intervention/follow up date?
- Tests/investigations in last three months
    - No
    - Yes: date and frequency of investigation
- Vaccinations
    - Flu vaccination in last year

   - Pneumococcal vaccination at least once since turning 65

3. Resource use questionnaire (to be extracted from care home records)

- Participant study ID
- Patient status (nursing / residential, noting any change in the past 6 months)
- In the past three months has the care home been the participant's only place of residence?
   - Yes
   - No – details of time spent in the care home and time spent elsewhere (days/months)
- Has the patient had any falls in the past three month?
- In the past 6 months has the participant had any other health service contact?
   - No
   - Yes – details of numbers of visit(s) or consultations, and with whom

Appendix 4. Inclusion of participants who lack capacity

1. Justification for the inclusion of participants who lack capacity (Adults with Incapacity AWI in Scotland)

Rationale for inclusion of participants who lack capacity (AWI participants) in this study.

i. Research evidence
ii. Generalisability of research findings
iii. CHIPPS study relates to treatment, not cause, of incapacity
iv. Scottish Government guidance

i. Research evidence

To exclude AWI will have implications for the robustness of the research, will not reflect everyday practice, and will exclude residents for whom a prescribing intervention may help address prescribing problems and thereby improve their health. These are described in more detail below.

In 2013, the Alzheimer's Society reported: (http://www.alz.co.uk/research/WorldAlzheimerReport2013ExecutiveSummary.pdf) that 80% of residents in care homes have some form of cognitive impairment. Therefore, to exclude AWI represents ignoring the majority of those in this setting, significantly limiting the generalisability of any research findings. Additionally, previous research has shown that this AWI population is frequently exposed to inappropriate prescribing of medication.[1] Therefore this is a group who are highly likely to benefit from this particular intervention.

Furthermore, there has been extensive research outlining the case of older adults, including those with incapacity, who have been systematically excluded from research. The seminal PREDICT study (Increasing the PaRticipation of the ElDerly In Clinical Trials) sought to investigate reasons for the exclusion of older people in research and to provide solutions for this problem. The researchers interviewed a range of older people with chronic conditions, including dementia, and in the case of the latter, participants stated that they could not understand why they were being excluded from research that is likely to be of direct benefit to them. A Health Technology Assessment report by Bartlett et al. entitled 'The causes and effects of socio-demographic exclusions from clinical trials' noted that exclusion was '*an injustice now being perpetuated against the old as regards the conduct of medical research*.'

ii. Generalisability

The research above provides evidence of the high number of AWI residents in care homes, it is necessary to include these residents to ensure the research results are generalisable to the whole population of residents in care homes, and not just to a minority of residents.

The CHIPPS WP6 protocol asks participating GPs to invite sufficient care home residents to enable recruitment of a minimum of 20 of their care home residents to participate in the study. The

protocol does not require GPs to exclude any residents at this stage who do not have capacity. Thus, on the basis of the Alzheimer's Society prevalence data above, it is likely that the cohort of invited residents will include a significant proportion of AWI.

If these residents were excluded the study would not be generalisable to care home populations and, assuming positive benefits of PIP involvement are found evidence would be generated to support a future PIP role for only the minority of care home patients.

Therefore, to generate evidence that PIPS can improve the care of patients in care homes, and also to ensure this is a robust definitive study it is crucial to include AWI residents.

iii. Research relates to treatment, not cause, of incapacity

The study is relating to the treatment of the person not the cause of the incapacity.

Although AWI are heterogeneous in terms of the cause of the incapacity, the incapacity often manifests itself in particular behaviours which are common across the group. These behaviours are often controlled with medication for the benefit of others and not to treat the individual patient - i.e. to reduce staff workload, or more intensive one-to-one interventions.

The CHIPPS study is aimed at making sure all prescribing is in the best interests of the patient - whether for mental or physical conditions - and also that all prescribing is appropriate.

Dementia is a leading cause of incapacity in older adults resident in care homes. In the year 2014/15, dementia accounted for the cause of incapacity in 84% of adults aged ≥ 65 years placed on welfare guardianship under the Adults with Incapacity (Scotland) Act 2000 (http://www.mwcscot.org.uk/media/240694/awi_monitoring_report__2__2014-15.pdf). It must be emphasised here that it is those patients with cognitive impairment, such as people with dementia (PWD), who represent an important group for whom the intervention – which aims to optimise prescribing - may benefit. In older people, a 'potentially inappropriate medication' (PIM) is any medication deemed to possess an unfavourable risk to benefit ratio, for example because it is associated with an increased risk of adverse side effects when used in older people. Indeed, research has shown that nearly half of PWD in care homes in England are prescribed at least one PIM, and medicines known as 'antipsychotics' account for the majority of such PIMs[1].

The over-use of these agents in PWD, has been recognised as a significant problem for decades, not only amongst healthcare professionals and researchers, but the wider public as well. However despite this awareness, inappropriate prescribing for this group persists and new approaches are required to address the problem. Currently, antipsychotics (as well as other psychoactive medicines such as sedatives) are being used inappropriately as 'chemical restraints'. Below are a selection of headlines (and links to the original articles), reported in the UK media in recent years, which illustrate the significant concern surrounding the inappropriate use of antipsychotics in PWD in care homes:

BBC News: ***"'Chemical cosh' dementia drug prescriptions concern"***

http://www.bbc.co.uk/news/health-13698487

The Irish Times: ***"Old and overmedicated: the drug problem in our nursing homes"***
http://www.irishtimes.com/news/social-affairs/old-and-overmedicated-the-drug-problem-in-our-nursing-homes-1.2232764

The Guardian: ***"Elderly in care 20 times more likely to be on antipsychotics"***
https://www.theguardian.com/society/2013/feb/21/elderly-care-antipsychotics-sharp-rise

The Scotsman: ***"Pharmacists fight 'chemical cosh' of elderly"***
http://www.scotsman.com/news/pharmacists-fight-chemical-cosh-of-elderly-1-1741739

Antipsychotics are associated with an increased risk of falls, cerebrovascular events, pneumonia and death in older adults[2]. A report published in 2009 by the Department of Health (http://www.dh.gov.uk/prod_consum_dh/groups/dh_digitalassets/documents/digitalasset/dh_108302.pdf) estimated that the use of antipsychotics results in 1,800 extra deaths and 820 serious adverse events (such as strokes) amongst PWD every year in the UK. This report called for a reduction in the usage of the antipsychotics in PWD to be made a clinical governance priority across the NHS.

This issue is of particular relevance to the CHIPPS programme, as it is known that PWD who live in care homes are *significantly more likely* to be prescribed antipsychotics than PWD who live at home. Previous initiatives have demonstrated that pharmacist-led medication reviews can lead to the reduction or discontinuation of inappropriate antipsychotic prescriptions in the majority of patients in a care home setting[3]. Tackling the over-use of antipsychotics, represents only one example of how PIPs can reduce inappropriate prescribing for PWD and cognitive impairment in care homes.

[1] Parsons C, Johnston S, Mathie E, Baron N, Machen I, Amador S, Goodman C. Potentially inappropriate prescribing in older people with dementia in care homes: A retrospective analysis. Drugs and Aging. 2012; 29: 143-55.

[2] Parsons C, Lapane K, Kerse N, Hughes C. Prescribing for older people in nursing homes: a review of the key issues. International Journal of Older People Nursing. 2011; 6: 45–54.

[3] Child A, Clarke A, Fox C, Maidment I. A pharmacy led program to review anti-psychotic prescribing for people with dementia. BMC Psychiatry. 2012; 12: 155.

iv. Scottish Government guidance

The Scottish Government guidance (http://www.gov.scot/Publications/2008/06/13114117/5) states that research, with adults incapable of consenting, is possible.

However, in order for research with adults incapable of consenting to be authorised, the guidance sets out, in 4.1 Section 51, all the circumstances and conditions which must apply.

Please see a summary of the points from this section of the guidance in Table 1 below, with evidence of their application in this study.

Table 1: Summary of points from Scottish Government guidance

	Summary of points in guidance	Does the study comply with this requirement?
1.	It will further knowledge	Yes<br><br>The CHIPPS study will provide knowledge about the effect of any treatment or care given to the adult; specifically it will provide evidence about whether a specially trained independent prescribing pharmacist (PIP), can improve the effectiveness of medications of elderly residents in care homes, which could be of significant value to the individual health and well-being of the resident. As AWI are likely to be a significant proportion of the resident cohort, and are known to be a group for whom prescribing is frequently inappropriate, it is important that they are included in the sample.
2.	It is of benefit to the adult or others in a similar condition	Yes<br><br>If the CHIPPS study suggested that a PIP's involvement is beneficial to the participating residents in the participating care home, then it is reasonable to assume this will benefit other care home residents. This definitive trial is designed to generate evidence for whether or not the service should be implemented more widely.<br><br>The CHIPPS study's inclusion criteria do not relate to a specific clinical condition but to the setting in which the adults are domiciled. The intervention is one which would apply to all patients regardless of condition and the outcome measures (which will show the effect of the

		intervention) are similarly selected to ensure they apply to a population with heterogeneous clinical conditions and also those with multiple conditions at an individual patient level.
3.	There is no unwillingness	Yes.<br><br>All patients will give signed consent, or if unable to give fully informed consent this will have been done on their behalf following a strict protocol (also see section below).<br><br>If an adult without capacity is recruited onto the study, the PIP will not proceed with delivering the intervention, if the resident shows any sign at all of unwillingness to the PIP being involved in their care.<br><br>The PIPs will have been specially trained by both a Consultant in Older Peoples Medicine and a Care of Older Person Specialist Pharmacist in how to work with elderly people in care homes, with and without capacity.
4.	The research has been approved by the appropriate Ethics Committee	Application has been made to the appropriate Ethics Committee.<br><br>Decision outstanding.
5.	It entails little or no risk or discomfort	Yes<br><br>The intervention of having a PIP sharing responsibility for residents' medications will involve no additional discomfort and is unlikely to pose any risk to participating residents additional to their usual care.

		All medicines have both benefits and risks and the patient is exposed to these whenever they take a medicine. Research already conducted with older patients with chronic pain suggests that when a pharmacist prescribes medicines rather than the doctor there are more side effects picked up and managed, pain is reduced, and patients are very satisfied with the service.<br><br>Bruhn H, Bond CM, Elliott AM, et al. Pharmacist led management of chronic pain in primary care: results from a randomised controlled exploratory trial. BMJ Open 2013;3:e002361.doi:10.1136/bmjopen-2012
6	Consent has been obtained from a person with relevant powers	Yes<br><br>There is a robust protocol in place to ensure that if a person without capacity is recruited, third party consent would have been obtained from a person with relevant powers. This involves contacting the resident's nearest relative, guardian or welfare of attorney and seeking their consent, on behalf of the resident.

Appendix 5. List of NCTU documents

NCTU approved documents only		
Document	v.	Date
CHIPPS WP6 CRF	2.0	(29.01.18)
SAE Form	1.1	5.1.18
Programme Steering Committee Terms of Reference	1.1	20.1.18
Data Monitoring Committee Terms of Reference	1.0	20.12.17
SAE Guidance	1.1	5.1.18
Safety Management Plan (SMP)	1.2	5.2.18
Trial quality management and monitoring plan (QMMP)	1.1	6.12.17
Risk Assessment (RA)	1.1	20.12.17

CHIPPS Logic model

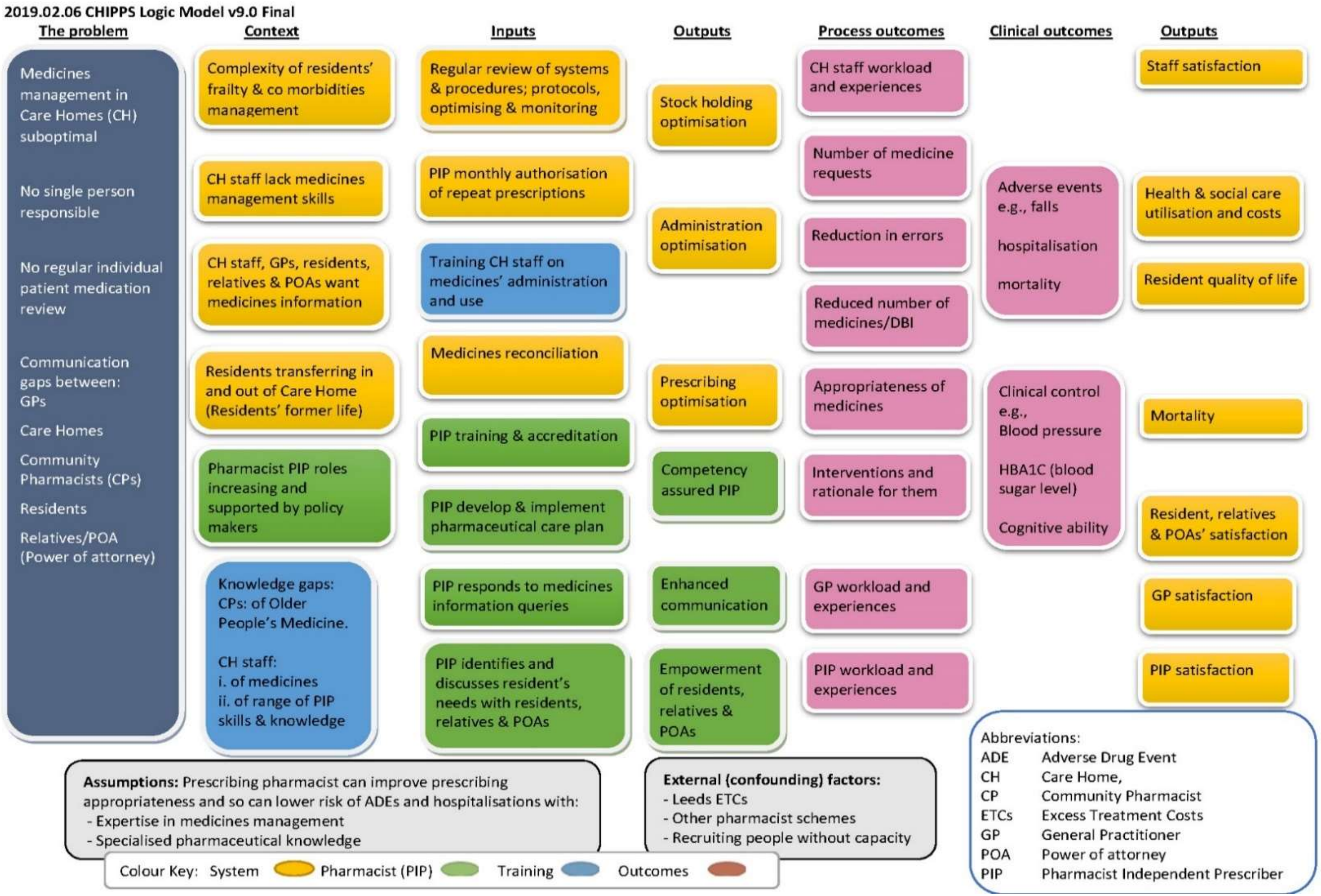

Supplementary File: Survival analysis comparing intervention with control

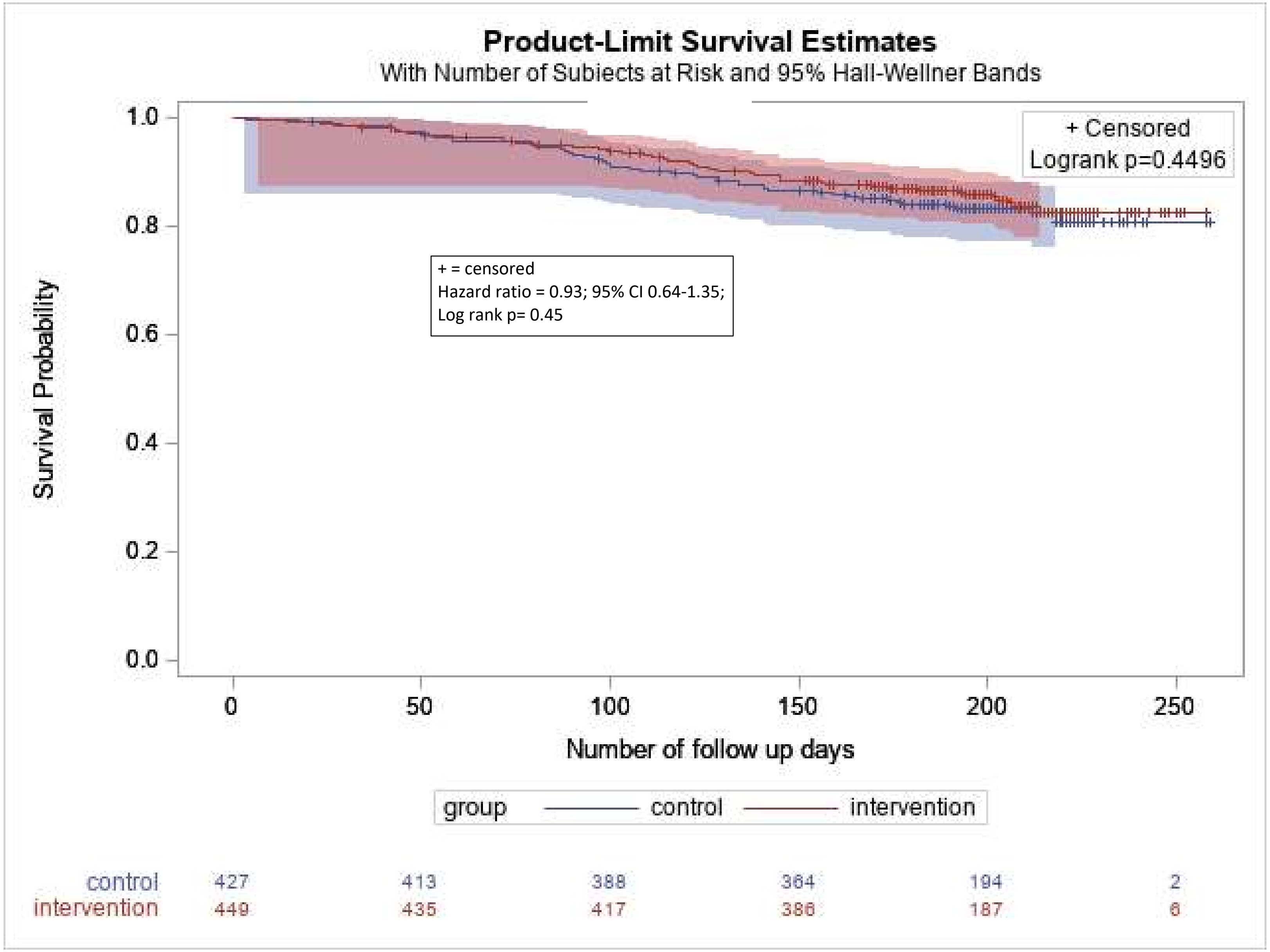

Supplement: Supplementary file 1 — Web appendix: Supplementary materials [file rich071883.ww1.pdf]
